# Mammary Adipocyte Size, Obesity-Related Breast Adipose Tissue Dysfunction, and Breast Cancer: A Systematic Review and Meta-Analysis

**DOI:** 10.3390/cancers18152464

**Published:** 2026-07-31

**Authors:** Ouafa Badre, Sue-Ling Chang, Belkacem Abdous, Julie Lemieux, Francine Durocher, André Tchernof, Caroline Diorio

**Affiliations:** 1 Department of Social and Preventive Medicine, Faculty of Medicine, Université Laval, Quebec City, QC G1V 0A6, Canada; ouafa.badre.1@ulaval.ca (O.B.); belkacem.abdous.1@ulaval.ca (B.A.); 2 Cancer Research Centre, Université Laval, Quebec City, QC G1J 0J9, Canada; sue-ling.chang@crchudequebec.ulaval.ca (S.-L.C.); julie.lemieux@crchudequebec.ulaval.ca (J.L.); francine.durocher@crchudequebec.ulaval.ca (F.D.); 3 Oncology Research Program, CHU de Québec–Université Laval Research Center, Quebec City, QC G1J 0J9, Canada; 4 Department of Medicine, Faculty of Medicine, Université Laval, Quebec City, QC G1V 0A6, Canada; 5 Center for Breast Diseases, Saint-Sacrement Hospital, CHU de Québec–Université Laval, Quebec City, QC G1S 4L8, Canada; 6 Endocrinology and Nephrology Research Program, CHU de Québec–Université Laval Research Center, Quebec City, QC G1V 4G2, Canada; 7 Department of Molecular Medicine, Faculty of Medicine, Université Laval, Quebec City, QC G1V 0A6, Canada; 8 School of Nutrition, Faculty of Agriculture and Food Sciences, Université Laval, Quebec City, QC G1V 0A6, Canada; andre.tchernof@criucpq.ulaval.ca; 9 Research Center, Quebec Heart and Lung Institute–Université Laval, Quebec City, QC G1V 4G5, Canada

**Keywords:** mammary adipocytes, adipocyte size, breast cancer, obesity, adipose tissue dysfunction, crown-like structures, aromatase, systematic review, meta-analysis

## Abstract

**Background/Objectives:** Mammary adipose tissue functions as an active endocrine organ, potentially influencing breast carcinogenesis through metabolic and inflammatory mechanisms. This systematic review synthesized evidence on associations between mammary adipocyte size, obesity-related adipose tissue dysfunction, and breast cancer. **Methods:** A comprehensive literature search was conducted across MEDLINE, EMBASE, CENTRAL, CINAHL and Web of Science (January 2010–September 2025) for observational studies measuring mammary adipocyte size in human breast tissue. Two reviewers independently conducted screening following the Cochrane Review’s rigorous methodology, and bias assessment using the ROBINS-E (Risk Of Bias In Non-randomized Studies of Exposure) tool. Random-effects meta-analyses were performed for quantifiable outcomes, with heterogeneity assessed using the *I*^2^ statistic. **Results:** Twenty-one studies were included in the systematic review. Meta-analyses indicated that mammary adipocyte diameter was positively correlated with body mass index (8 studies; *n* = 720; correlation coefficient (*r*) = 0.44, 95% CI: 0.29–0.56), crown-like structure (CLS) density (5 studies; *n* = 383; *r* = 0.45, 95% confidence interval (CI): 0.36–0.54), aromatase expression (3 studies; *n* = 333; *r* = 0.36, 95% CI: 0.05–0.61), and three studies compared adipocyte diameter between CLS-positive and CLS-negative tissue (*n* = 297; mean difference = 9.69 μm, 95% CI: 4.94–14.44). **Conclusions:** This review summarizes moderate positive correlations of mammary adipocyte size with systemic adiposity, local breast inflammation, and aromatase expression, supporting a potential role for mammary adipose tissue in obesity-related metabolic dysfunction. Future prospective studies with standardized measurement protocols and improved control for confounding variables are needed to strengthen causal inference and clinical applications.

## Simple Summary

Breast tissue contains fat cells that do more than store energy, as they actively produce hormones and inflammatory signals that may influence breast cancer development. As obesity rates rise globally, understanding how the size of these fat cells relates to metabolic disturbances and breast cancer has become increasingly important. This systematic review and meta-analysis aimed to synthesize existing evidence on whether larger breast fat cells are associated with obesity, local breast inflammation, and increased estrogen production within breast tissue. Our analyses revealed positive associations between breast fat cell size and body weight, local inflammation, and the activity of an enzyme responsible for local estrogen production. These findings suggest that breast fat tissue plays an active biological role in processes that may promote breast cancer and highlight the need for standardized measurement approaches in future research to better guide prevention and treatment strategies.

## 1. Introduction

Breast cancer is the most diagnosed cancer and remains the leading cause of cancer-related mortality among women worldwide, with over 2.3 million new cases and around 685,000 deaths reported in 2020 [1]. Despite significant advances in screening and treatment modalities, its global burden continues to increase [2,3], underscoring the critical importance of identifying potentially modifiable risk factors associated with its development and progression.

Among these factors, obesity and its associated metabolic disturbances have received increasing attention. Excess adiposity, often accompanied by features of metabolic syndrome such as insulin resistance, hypertension, hyperglycemia, and dyslipidemia, is associated with elevated breast cancer incidence and adverse clinical outcomes, particularly among postmenopausal women [4,5,6], through pathways involving insulin-like growth factor 1 (IGF-1) signaling and chronic low-grade systemic inflammation [4,6,7,8,9]. Obesity is itself an established risk factor for postmenopausal breast cancer and is linked to poorer prognosis, an effect that appears to depend on menopausal status and to be driven partly by adipose tissue dysfunction rather than by excess fat mass alone [10].

In this metabolic paradigm, mammary adipose tissue has emerged as an active endocrine and paracrine organ involved in breast carcinogenesis, particularly in the setting of obesity. Breast microenvironment adipocytes secrete various adipokines, inflammatory cytokines, and growth factors that may promote tumor development and progression [4,11,12]. Among women with obesity, adipocyte hypertrophy throughout the mammary tissue is associated with local chronic inflammation, increased aromatase expression, and dysregulated estrogen biosynthesis, leading to an estrogen-rich, pro-tumorigenic environment [13,14]. This dysfunctional state also involves hypoxia, insulin resistance, and an imbalance in adipokine secretion, which together reinforce a microenvironment conducive to tumor initiation and growth [10]. Beyond these tissue-wide alterations, adipocytes located at the invasive tumor margin undergo a further transformation upon direct contact with cancer cells, giving rise to so-called cancer-associated adipocytes [15]. These adipocytes lose part of their lipid content and mature differentiation markers, adopt a fibroblast-like appearance, and shift their secretory activity toward pro-inflammatory cytokines and tumor-supportive factors [16]. Through a reciprocal exchange of signals with adjacent tumor cells, cancer-associated adipocytes contribute to extracellular matrix remodeling, sustained aromatase activity, and metabolic reprogramming, thereby favoring tumor invasion and progression [17]. Within this context, mammary adipocyte size has been proposed as a directly measurable morphological marker of adipose tissue dysfunction, which may reflect the combined effects of hypertrophy, local inflammation, and altered estrogen production [18].

However, while some studies have investigated the relationship between mammary adipocyte size, adipose tissue dysfunction, and breast cancer [17,19], the available evidence remains disparate and inconsistent. Variability in study designs, methods for measuring adipocytes, and study populations limits the ability to draw definitive conclusions about the clinical significance of these associations.

Given this biological background, we hypothesized that larger mammary adipocyte size may reflect underlying adipose tissue dysfunction and would therefore be associated with greater systemic adiposity, more pronounced local breast inflammation, and more aggressive breast cancer characteristics, features that together characterize a tumor-promoting breast microenvironment. Therefore, this systematic review aims to synthesize and critically evaluate available evidence, in adult women providing human breast tissue samples, on the association between mammary adipocyte size and adiposity, local inflammation, metabolic dysfunction, and breast cancer features.

## 2. Materials and Methods

This systematic review and meta-analysis was conducted following the recommendations of the Cochrane handbook for systematic reviews of interventions [20] and reported according to the Preferred Reporting Items for Systematic Reviews and Meta-Analyses (PRISMA) 2020 guidelines and has not been registered [21]. The PRISMA checklist can be found in the Supplementary File. As this study was based on the collection and synthesis of data from published articles, ethical approval was not required.

### 2.1. Search Methods for Identification of Studies

A systematic literature search was conducted in five electronic databases: MEDLINE (via PubMed), EMBASE, the Cochrane Central Register of Controlled Trials (CENTRAL), CINAHL and Web of Science. The search covered studies published between January 2010 and September 2025. This lower temporal bound reflects the emergence of the specific field addressed in this review, as the quantitative histological assessment of mammary adipocyte size in human breast tissue in relation to local inflammation, aromatase expression, and tumor characteristics developed essentially from 2011 onward, following the founding description of crown-like structure in the human breast environment [14] and of cancer-associated adipocytes [22]; studies predating this period would therefore not meet the eligibility criteria.

The search strategy was developed using a combination of Medical Subject Headings (MeSH) terms and free-text keywords related to mammary adipocyte size, obesity, metabolic dysfunction, local inflammation, and breast cancer. Filters were applied to exclude animal studies, with no language restrictions. Additionally, the reference lists of all included articles were manually reviewed to identify relevant studies that may not have been captured by electronic search. The complete search strategies are available in the Supplementary Materials (Table S1).

### 2.2. Criteria for Considering Studies for This Review

#### 2.2.1. Types of Studies

Eligible studies included observational designs, such as cross-sectional, case–control, and cohort studies, as well as clinical trials that measured mammary adipocyte size as part of the study protocol. Studies were excluded if they involved in vitro or animal models, or if they were conference abstracts, letters, or editorials that lacked original data.

#### 2.2.2. Types of Participants

Studies enrolling adult human participants (aged ≥ 18 years), regardless of sex or ethnicity, were eligible for inclusion. The analyzed tissue had to be derived from human breast samples, collected either prior to or following a breast cancer diagnosis.

#### 2.2.3. Types of Exposure

The exposure of interest was the size of mammary adipocytes, as measured in histological sections using either conventional microscopy or digital image analysis software (e.g., ImageJ, National Institutes of Health, Bethesda, MD, USA; HALO Indica Labs, Albuquerque, NM, USA; or equivalent tools). Adipocyte measurements could be obtained from tumor-adjacent (peritumoral) or tumor-free mammary tissue.

#### 2.2.4. Types of Outcomes

Eligible outcomes included body mass index (BMI), calculated from measured or self-reported height and weight; obesity and metabolic dysfunction and its components (e.g., diabetes, hypertension, dyslipidemia, and waist circumference or waist-to-hip ratio, assessed using standard anthropometric protocols where reported); local inflammation in breast adipose tissue (e.g., crown-like structures (CLS), macrophage infiltration), assessed by immunohistochemistry; systemic and tissue-level biomarkers (e.g., high-sensitivity C-reactive protein (hsCRP), interleukin-6 (IL-6), leptin, adiponectin, aromatase expression (CYP19A1), and hydroxysteroid dehydrogenase (HSD)), quantified by immunoassay or gene expression analysis; and breast cancer prognostic factors (e.g., histologic type, tumor grade, molecular subtype, and survival outcomes).

### 2.3. Selection of Studies

The selection of studies was conducted in two stages. First, the titles and abstracts of all retrieved references were screened to exclude clearly irrelevant articles. Then, the full texts of potentially eligible studies were reviewed to assess inclusion based on predefined eligibility criteria. Two reviewers (OB and CD) independently screened all records using Covidence software (Veritas Health Innovation, Melbourne, Australia; available at https://www.covidence.org), and any disagreements were resolved through discussion.

### 2.4. Data Extraction

Data extraction was carried out using a standardized and piloted data extraction form developed for this review. Extracted information included study design, characteristics of participants, adipocyte measurement methods, exposure variables, adjustments for confounding factors, and reported effect measures. PlotDigitizer software (version 3.1.6; available at https://plotdigitizer.com) was used to extract quantitative values from figures and plots when numerical data were available only in graphical format [23]. In cases where multiple publications referenced the same study population, the most comprehensive or recent source was prioritized, and information was supplemented by secondary reports as necessary. Data were independently extracted (OB) on two separate occasions to ensure consistency and accuracy.

### 2.5. Risk of Bias Assessment in Retained Studies

The risk of bias in the included studies was evaluated using the ROBINS-E (Risk of Bias in Non-randomized Studies of Exposure) tool, updated in 2024 [24]. One reviewer (OB) assessed each study across key domains, including participant selection, exposure classification, measurement of outcomes, handling of confounding variables, missing data, and selective reporting. When necessary, a second reviewer (CD) was consulted to resolve uncertainties or confirm decisions.

### 2.6. Assessment of Heterogeneity

Sources of heterogeneity were examined by looking at differences in study design, population characteristics, adipocyte measurement techniques, and outcomes assessed. Statistical heterogeneity was evaluated using the *I*^2^ statistic and Cochran’s *Q* test. In addition, given that methodological variability in adipocyte measurement may represent a key source of heterogeneity in this field, a subgroup analysis was conducted, when applicable (i.e., when a sufficient number of studies was available), stratifying studies by adipocyte measurement method (manual versus digital/software-assisted) to assess its contribution to the observed heterogeneity. Planned sensitivity analyses were conducted to assess the robustness of the findings concerning methodological quality and exposure measurement.

### 2.7. Data Synthesis

Confidence intervals for Spearman correlation coefficients were calculated using the Bonett & Wright (2000) correction [25]. A random-effects model was used for the meta-analysis to account for variability among studies and provide a more accurate estimate of the overall effect [26]. The results were presented using forest plots, which visualize point estimates and their corresponding confidence intervals. Publication bias was assessed both visually through funnel plots and statistically using Egger’s regression test [27]. All analyses were performed using R version 4.3.1 (R Foundation for Statistical Computing, Vienna, Austria) with the *meta* (version 8.1-0) and *metafor* (version 4.8-0) packages.

### 2.8. Sensitivity Analysis

Sensitivity analyses were pre-specified to assess the robustness of the results. First, a leave-one-out method was applied to examine the influence of each study on the overall results. Next, a secondary analysis was conducted by excluding studies for which effect sizes were estimated indirectly from published graphs using PlotDigitizer software, as these estimates may be subject to measurement error. The goal was to determine whether including these approximated data affected the overall findings and the degree of heterogeneity.

## 3. Results

### 3.1. Study Selection

**Figure 1:**
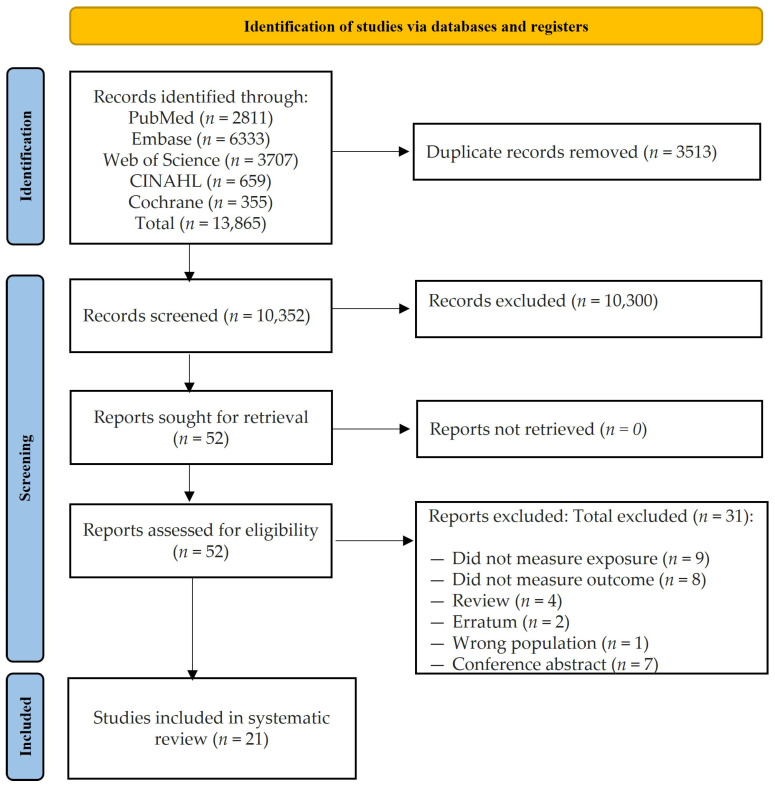
Flow diagram of study selection according to PRISMA guidelines. The selection process includes four main stages: identification of studies in databases, initial screening, eligibility assessment, and final inclusion in the systematic review. Numbers in parentheses indicate the number of studies at each stage. PRISMA = Preferred Reporting Items for Systematic Reviews and Meta-Analyses.

The search identified 13,865 records across five databases. After removing duplicates, 10,352 unique records were screened by title and abstract. A total of 52 articles were assessed in full text. Of these, 31 were excluded due to the absence of adipocyte size measurement, absence of relevant outcomes, non-eligible study design, or duplicate data. In total, 21 studies were included in the systematic review. The full selection process is summarized in Figure 1.

### 3.2. Study Characteristics

Twenty-one studies were included in this review. Most studies used a cross-sectional design (*n* = 12), while four were retrospective, two were prospective observational, two were nested case-controls, and one was a self-controlled paired study. Sample size ranged from 10 to 347 participants. Most studies were hospital-based (*n* = 18), with limited longitudinal follow-up (*n* = 3). Studies were conducted primarily in North America (*n* = 13) and Europe (*n* = 6), with fewer from Asia (*n* = 2) and Mexico (*n* = 1).

Participants’ mean age ranged from 43.4 to 70.3 years, and mean BMI from 22 to 37 kg/m^2^ (*n* = 19). Waist circumference and waist-to-hip ratio (WHR) were reported in only two studies. Most studies included both pre- and postmenopausal women (*n* = 17). Breast cancers were predominantly invasive carcinoma (*n* = 15), with estrogen receptor (ER)-positive tumors ranging from 63% to 100%, progesterone receptor (PR)-positive from 52% to 89%, and human epidermal growth factor receptor 2 (HER2)-positive from 5% to 29%. Tumors were mostly T1/T2 stage, grade 2 or 3, with early-stage disease (I/II) predominant.

Adipocyte size was evaluated by diameter in 18 studies and by area in 6 studies, with one study measuring only adipocyte number. Digital image analysis was employed in 17 studies, while 4 used manual measurements, most commonly at 20× magnification (*n* = 10). The number of adipocytes measured per sample ranged from 18 to 250 cells across studies, with 30 cells being the most frequently applied threshold (*n* = 5); only one study reported a formal measurement reproducibility metric (intraclass correlation coefficient ICC = 0.89) [28]. Mean adipocyte diameters ranged from 64 to 113 µm. Tissue was obtained from mastectomy (*n* = 9), mixed procedures (*n* = 5), tumorectomy (*n* = 2), or unreported procedures (*n* = 5). Circulating inflammatory markers (hsCRP, IL-6, leptin) were measured in nine studies, while tissue inflammation markers (CLS density, tumor-associated macrophages (TAM), IL-6, monocyte chemoattractant protein-1 (MCP-1), and tumor necrosis factor (TNF)) were assessed in 12 studies. Metabolic markers (insulin, diabetes, hypertension, liver enzymes, metabolic syndrome (MetS) criteria, and aromatase) were evaluated in four studies. HSD expression in the breast was measured in one study and circulating sex hormone-binding globulin (SHBG) levels in one study (detailed characteristics are presented in Table S2).

### 3.3. Risk of Bias in Included Studies

**Figure 2:**
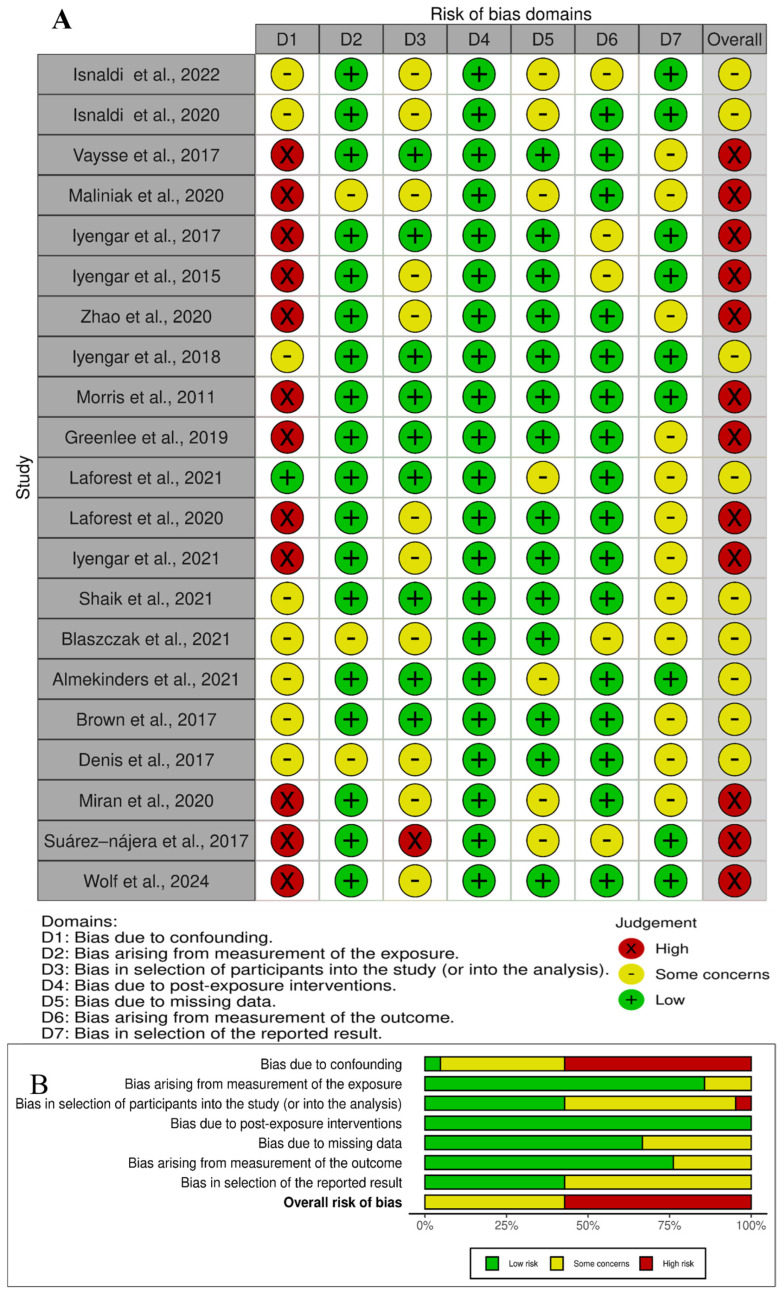
Risk of bias assessment for included studies using ROBINS-E tool [14,19,28,29,30,31,32,33,34,35,36,37,38,39,40,41,42,43,44,45,46]. (**A**) Individual study assessment across seven domains (D1–D7). (**B**) Summary distribution across all domains. Green indicates low risk, yellow indicates some concerns, and red indicates high risk of bias. ROBINS-E = Risk Of Bias In Non-randomized Studies of Exposure.

Most of the included studies were assessed to have either a high overall risk of bias (*n* = 12) or some concerns (*n* = 9) (Figure 2). Studies rated as high risk predominantly showed critical issues with confounding, while studies with some concerns generally had moderate issues with confounding, selection bias, and reporting bias. In contrast, the risk of bias in measuring exposures and outcomes, post-exposure interventions, and missing data was generally low. A detailed assessment of each study is presented in Figure 2.

### 3.4. Mammary Adipocyte Size and Adiposity Indicators

Twelve studies examined associations between mammary adipocyte size and adiposity indicators, demonstrating positive correlations with BMI (*n* = 12), waist circumference (*n* = 1) and WHR (*n* = 2), and an inverse association between adipocyte number and BMI (*n* = 2).

#### 3.4.1. Quantitative Synthesis: Correlation Between Mammary Adipocyte Diameter and BMI

**Figure 3:**
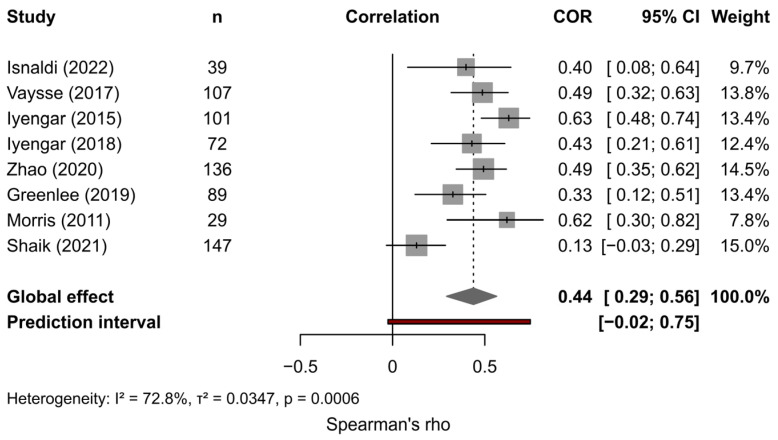
Forest plot of the correlation between mammary adipocyte diameter and BMI. Individual study Spearman correlation coefficients with 95% confidence intervals and pooled estimate using a random-effects model. The diamond represents the overall effect size [14,19,33,34,35,36,37,38]. Substantial heterogeneity was detected (*I*^2^ = 72.8%). BMI = body mass index.

Twelve studies evaluated the correlation between the diameter of mammary adipocyte size and BMI. Four studies were not included in the quantitative synthesis due to insufficient or non-comparable data [29,30,31,32], leaving eight studies (720 participants) eligible for meta-analysis [14,19,33,34,35,36,37,38]. All included studies reported Spearman correlation coefficients. Based on a random-effects model, a moderate positive overall correlation was observed (Spearman’s *r* = 0.44; 95% CI: 0.29–0.56) (Table S3A) (Figure 3).

Substantial heterogeneity was observed among the studies (*I*^2^ = 72.8%, *Q* = 25.69, *p* < 0.0001) (Figure 3). Leave-one-out analysis confirmed the robustness of the overall estimate, with correlations ranging from *r* = 0.42 to *r* = 0.49, while identifying a single study [33] as the main contributor to this heterogeneity (Figure S1). Its exclusion markedly reduced heterogeneity (*I*^2^ = 26.5%, *Q* = 8.16, *p* = 0.0548) and yielded a slightly stronger correlation (*r* = 0.48; 95% CI: 0.38–0.59) (Figures S2 and S3). This study differed from the others in the tissue analyzed: adipocytes were assessed largely in benign breast tissue rather than in tissue from patients with diagnosed breast cancer, which plausibly explains its outlying effect. Excluding two studies [19,38] with reconstructed correlation coefficients from digitized graphs yielded a similar overall correlation (*r* = 0.45; 95% CI: 0.24–0.62) (Figures S4 and S5), and did not account for the observed heterogeneity.

Subgroup analysis by measurement method showed no significant difference between studies using manual measurement (*r* = 0.50; 95% CI: 0.22–0.70; *n* = 4) and those using digital/software-assisted measurement (*r* = 0.38; 95% CI: 0.08–0.62; *n* = 4; test for subgroup differences: *χ*^2^_1_ = 1.07, *p* = 0.301) (Figure S6). The higher heterogeneity in the digital/software-assisted subgroup (*I*^2^ = 78.7%) was driven entirely by Shaik (2021) [33]: after its exclusion, the two subgroups became nearly identical (manual, *r* = 0.50; digital/software-assisted, *r* = 0.48), with no residual heterogeneity in the digital subgroup (*I*^2^ = 0%), and the difference between subgroups remained non-significant (*χ*^2^_1_ = 0.07, *p* = 0.796) (Figure S7; Table S3B). Adipocyte measurement method therefore did not account for the observed heterogeneity.

**Figure 4:**
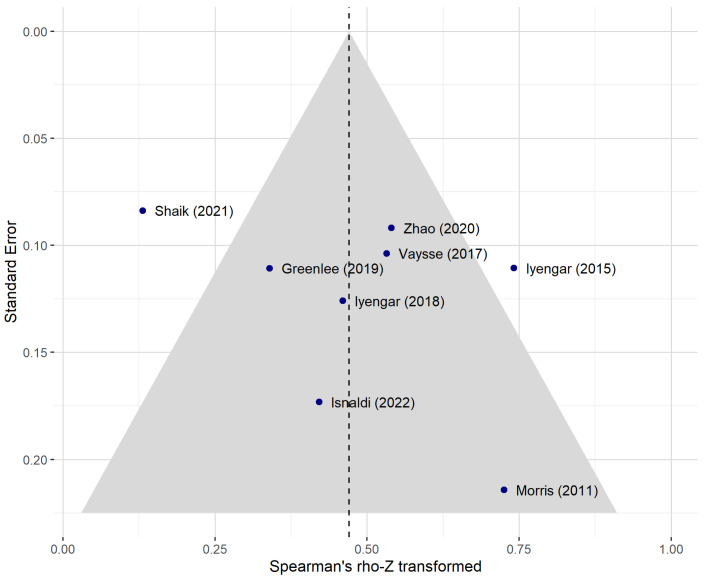
Funnel plot assessing publication bias in the correlation between mammary adipocyte diameter and BMI [14,19,33,34,35,36,37,38]. Fisher’s Z-transformed correlation coefficients (with Bonett & Wright correction) plotted against standard error [25]. Each point represents one study. The vertical dashed line indicates the pooled Fisher’s Z-transformed correlation coefficient. The distribution appears broadly symmetrical, and Egger’s test showed no significant evidence of publication bias. BMI = body mass index.

Visual inspection of the funnel plot and Egger’s test (*t* = 1.12, *p* = 0.305) revealed no significant evidence of publication bias or small-study effects (Figure 4). Nevertheless, the trim-and-fill method imputed three studies on the lower-correlation side, yielding an attenuated but still positive adjusted estimate (*r* = 0.34; 95% CI: 0.16–0.49) (Figure S8).

#### 3.4.2. Narrative Synthesis: Associations Between Mammary Adipocyte Size and Adiposity Indicators

Across two independent cohorts, mean adipocyte diameter was consistently larger in the higher BMI category (<25 vs. ≥25 kg/m^2^: 60.4 vs. 69.0 µm, *p* = 0.012 [19]; 86.5 vs. 95.8 μm, *p* < 0.001 [37]), and a progressive gradient across three BMI categories (18.5–24.9/25.0–29.9/≥30.0 kg/m^2^) in an exploratory cohort (*n* = 10) confirmed this trend for both diameter (τ = 0.59–0.75) and area (τ = 0.65–0.80), more markedly in cancer-associated than in distant adipocytes [31]. This gradient was tissue-specific: adipocyte area rose with BMI in the tumor-proximal adipocyte row (1541 vs. 2377 µm^2^, *p* = 0.019) and the tumor-distal row (2345 vs. 3849 µm^2^, *p* = 0.037), but not in peripheral tissue (*p* = 0.067) [30,31].

Beyond BMI, waist circumference categories (<80, 80–88, ≥88 cm) and waist-to-hip ratio categories (≤0.80, 0.80–0.85, ≥0.85) showed stepwise increases in adipocyte diameter (68.5, 79.1, 86.3 μm and 71.1, 83.2, 86.1 μm respectively, both *p* < 0.0001) [28], though another study found borderline significance for waist-to-hip ratio (*p* = 0.057) [19]. Adipocyte diameter also correlated with body fat percentage (*r* = 0.52, *p* < 0.01) [36], dual-energy X-ray absorptiometry (DXA)-measured truncal fat (*p* < 0.001) [19] and breast non-dense area (β = 0.10 μm per cm^2^, *p* < 0.0001) [28].

However, adipocyte number showed the opposite pattern, decreasing with higher BMI (*r* = −0.47, *p* < 0.001 [32]; BMI <25: 341 vs. BMI ≥25: 257 adipocytes, *p* < 0.001 [19]). Longitudinally, adipocyte size was positively associated with BMI at age 18 (*p* = 0.0456) and change in BMI over time (*p* < 0.0001), while showing an inverse association with birth weight (*p* = 0.0349) [28] (Table S3A).

### 3.5. Mammary Adipocyte Size and Inflammatory Markers

Fourteen studies examined associations between mammary adipocyte size and inflammatory markers, demonstrating positive correlations with crown-like structure (CLS) density (*n* = 9), an inverse association between adipocyte number and CLS density (*n* = 1), larger adipocytes in CLS-positive (*n* = 7) and inflamed tissue (*n* = 2), positive associations with systemic inflammatory biomarkers (hsCRP, IL-6, leptin) (*n* = 2) and gene expression markers (IL-6, leptin (LEP), TNF) (*n* = 1), an inverse association with adiponectin (*n* = 1), and no significant associations with tumor-associated macrophages (*n* = 2), infiltrating lymphocytes (*n* = 1), or IL1B expression (*n* = 1).

#### 3.5.1. Quantitative Synthesis: Correlation Between Mammary Adipocyte Diameter and Crown-like Structure Density

**Figure 5:**
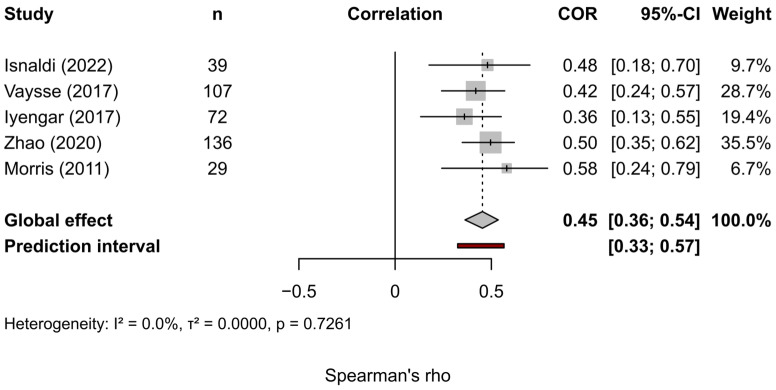
Forest plot of the correlation between mammary adipocyte diameter and crown-like structure density [14,19,34,37,39]. Individual study Spearman correlation coefficients with 95% confidence intervals and pooled estimate using a random-effects model. The diamond represents the overall effect size. No heterogeneity detected (*I*^2^ = 0%).

Five studies (*n* = 383) were included in the meta-analysis examining the correlation between crown-like structure (CLS) density and mammary adipocyte diameter [14,19,34,37,39], all reporting Spearman correlation coefficients. The overall correlation was moderate and positive (*r* = 0.45; 95% CI: 0.36–0.54) (Table S3B) (Figure 5).

No heterogeneity was observed (*I*^2^ = 0%, *Q* = 2.05, *p* = 0.7261). The leave-one-out analysis showed that the pooled correlation remained consistent (*r* range: 0.44–0.47), indicating that two studies [14,19] with coefficients estimated from digitized graphs yielded a similar correlation (*r* = 0.45; 95% CI: 0.26–0.62), with unchanged heterogeneity (*I*^2^ = 0%, *p* = 0.55) (Figures S9–S11).

**Figure 6:**
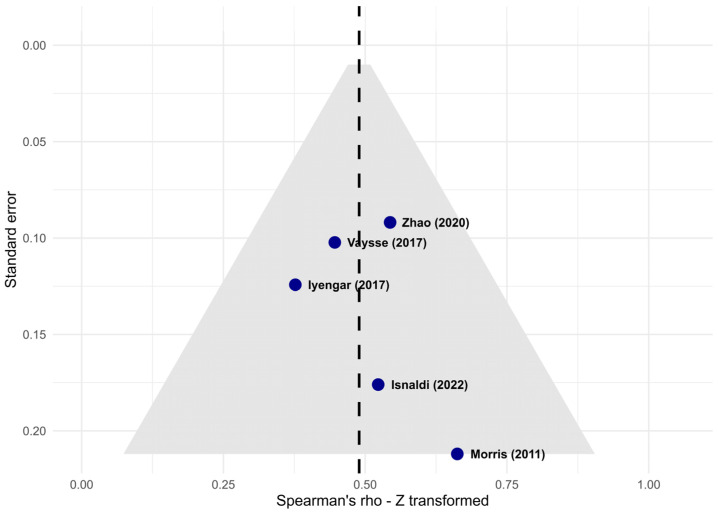
Funnel plot assessing publication bias in the correlation between mammary adipocyte diameter and crown-like structure density [14,19,34,37,39]. Fisher’s Z-transformed correlation coefficients (with Bonett & Wright correction) plotted against standard error [25]. Each point represents one study. The vertical dashed line indicates the pooled Fisher’s Z-transformed correlation coefficient. The symmetrical distribution suggests no evidence of publication bias.

Funnel plot inspection and Egger’s test (*t* = 0.41, *p* = 0.684) showed no publication bias, and the trim-and-fill method identified only one potentially missing study with minimal impact on the overall results (Figure 6 and Figure S12).

#### 3.5.2. Quantitative Synthesis: Comparison of Mammary Adipocyte Diameter Between CLS-Positive and CLS-Negative Tissue

**Figure 7:**
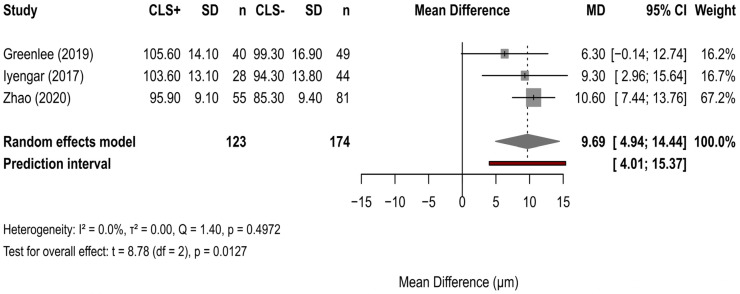
Forest plot of mean difference in mammary adipocyte diameter between CLS-positive and CLS-negative mammary tissue [37,38,39]. Individual study mean differences with 95% confidence intervals and pooled estimate using random-effects model. Diamond represents overall effect size. No heterogeneity detected (*I*^2^ = 0%). CLS = crown-like structures; MD = mean difference.

Three studies (*n* = 297) compared mammary adipocyte diameter between tissues with CLS presence and CLS absence [37,38,39]. The pooled mean difference showed that adipocytes in tissue with CLS were significantly larger, with a mean difference of 9.69 μm (95% CI: 4.94–14.44, *p* = 0.0127). No heterogeneity was detected across studies (*I*^2^ = 0%, *p* = 0.4972) (Figure 7).

Leave-one-out sensitivity analysis confirmed the stability of results, with pooled mean differences ranging from 7.4 to 10.6 μm (Figure S13).

**Figure 8:**
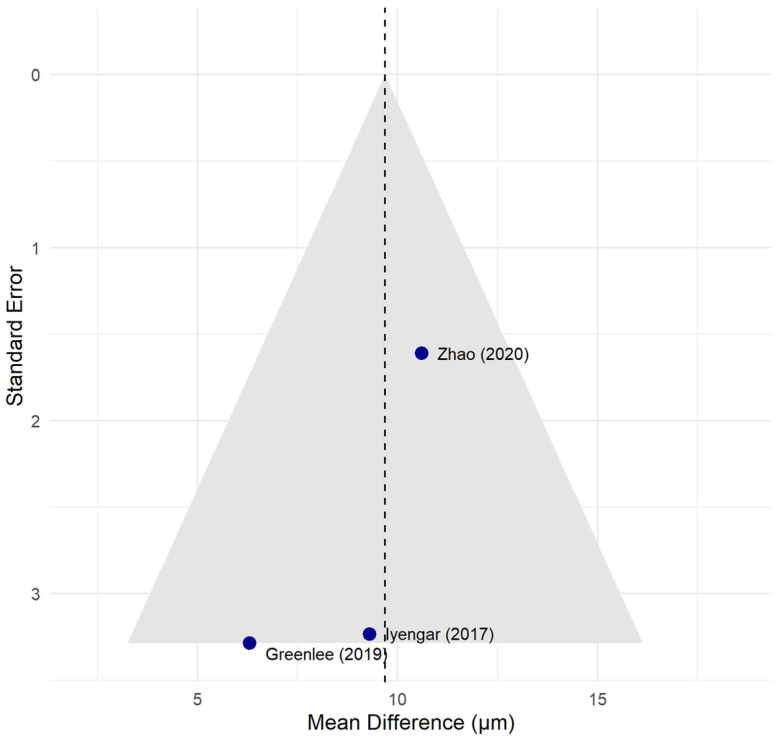
Funnel plot assessing publication bias in studies comparing mammary adipocyte diameter between CLS-positive and CLS-negative mammary tissue [37,38,39]. Each point represents individual study plotted by mean difference against standard error. The vertical dashed line indicates the pooled mean difference. Symmetrical distribution suggests low risk of publication bias. CLS = crown-like structures.

Egger’s test (*t* = −1.61, *p* = 0.354) indicated no significant publication bias. Trim-and-fill analysis identified two potential missing studies (Figure 8 and Figure S14).

#### 3.5.3. Narrative Synthesis: Associations Between Mammary Adipocyte Size and Inflammatory Markers

Studies examining adipocyte area and CLS density showed positive correlations (*r* = 0.47, *p* < 0.001 for area at 75th percentile; *r* = 0.48, *p* = 0.004 for mean area in distant tissue) [34,40], although one small preliminary study (*n* = 12) found this association only in unaffected breasts (*r* = 0.65, *p* = 0.032) and not in affected ones (*r* = 0.26, *p* = 0.497) [29]. Research examining CLS presence consistently showed larger adipocytes in CLS-positive tissue (*p* = 0.001 to 0.023) [19,33,34], with mean diameter differences of 15 μm (*p* < 0.001) [35], while adipocyte number showed a non-significant inverse trend with CLS density (*r* = −0.18, *p* = 0.12) [32]. Additionally, significant associations between adipocyte size and CLS presence were found in univariable regression (β = 0.79 for area, *p* = 0.023; β = 0.81 for diameter, *p* = 0.021) but became non-significant after BMI adjustment (β = 0.59 for area, *p* = 0.083; β = 0.61 for diameter, *p* = 0.077) [34] (Table S3B).

In contrast, tumor-associated macrophage (TAM) density showed only non-significant trends with mean adipocyte diameter (β = 0.71, *p* = 0.078) and area (β = 0.70, *p* = 0.075), which remained non-significant after BMI adjustment (*p* = 0.122 and *p* = 0.113, respectively) [34], and infiltrating lymphocytes showed a comparable trend (*p* = 0.051) [33]. One exception was reported for M1 (pro-inflammatory) and total macrophage counts, which correlated positively with adipocyte diameter after adjustment for age and menopausal status (*p* < 0.001) [28] (Table S3B).

Similarly, studies examining breast white adipose tissue inflammation (BWATi) consistently showed larger adipocyte diameters in inflamed tissue. Women with severe BWATi had significantly larger mean and median adipocyte diameters compared to those without inflammation (108.3 µm vs. 99.3 μm, difference 9.0 μm, *p* = 0.04; and 93.9 μm vs. 85.8 μm, difference 8.1 μm, *p* < 0.001, respectively) [38,41]. Mild BWATi showed no significant difference (*p* = 0.31) [38]. Notably, the association persisted in women with BMI below 25 kg/m^2^ (95.8 μm vs. 85.6 μm, median difference 10.2 μm, *p* = 0.024) [41].

Regarding systemic inflammatory biomarkers, two studies showed that hsCRP had positive associations with adipocyte diameter (*r* = 0.50 and *r* = 0.47, respectively, *p* < 0.01) [36,41]. Furthermore, IL-6 (*r* = 0.47, *p* < 0.001) and leptin (*r* = 0.56, *p* < 0.001) also demonstrated positive correlations with adipocyte diameter, while adiponectin showed an inverse association (*r* = −0.33, *p* < 0.001) [41] (Table S3B).

At the gene expression level, one study examining inflammatory markers in mammary adipose tissue reported significant differences in adipocyte diameter between high and low expression groups for interleukin-6 (IL-6), leptin (LEP), and TNF, with mean differences of 8.1, 10.3, and 9.4 μm, respectively (*p* < 0.001), after adjusting for age and menopausal status [28]. No association was found with interleukin-1 beta (IL1B) expression (*p* = 0.631) (Table S3B).

### 3.6. Mammary Adipocyte Size and Metabolic Biomarkers

Six studies examined associations between mammary adipocyte size and metabolic biomarkers [28,38,39,41,42,43]. Larger adipocytes were associated with diabetes (*n* = 2), elevated insulin (*n* = 1) and alanine aminotransferase (ALT) levels (*n* = 1), MetS (*n* = 1), and hypertension (*n* = 1), with no association for aspartate aminotransferase (AST) (*n* = 1). Also, a moderate positive correlation was observed with aromatase expression (*n* = 4), as well as positive associations with HSD11B1 expression (*n* = 1), while inverse associations were observed with SHBG levels (*n* = 1) and HSD17B12 expression (*n* = 1), and no significant associations with HSD17B7 (*n* = 1) or peroxisome proliferator-activated receptor gamma PPARG expression (*n* = 1).

#### 3.6.1. Associations Between Mammary Adipocyte Size and Metabolic Dysfunction Markers

Four studies examined the association between mammary adipocyte size and metabolic markers [28,38,41,42]. Adipocyte area was significantly higher in women with diabetes than in those without (4744 vs. 3198 μm^2^, *p* = 0.008) [42], while another study reported a similar but non-significant pattern (86.3 vs. 78.5 μm, *p* = 0.070) [28].

Adipocyte diameter correlated positively with serum ALT levels (*p* < 0.050) but showed no association with AST (*p* = 0.170) [38]. A positive correlation was also observed between adipocyte diameter and insulin levels (*r* = 0.40) [41].

Adipocyte diameter increased progressively with the number of metabolic syndrome criteria; women meeting four criteria had adipocytes 14.7 μm larger than those with none (*p* = 0.004) [28]. Larger adipocytes were also observed among women with hypertension (85.3 vs. 77.1 μm, *p* = 0.001) [28].

#### 3.6.2. Associations Between Mammary Adipocyte Size and Other Metabolic Biomarkers

##### Quantitative Synthesis: Association Between Mammary Adipocyte Diameter and Aromatase Expression

**Figure 9:**
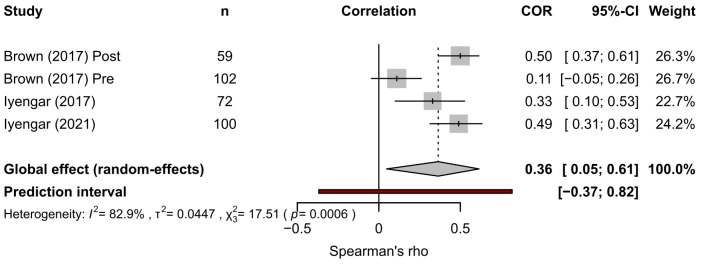
Forest plot of correlation between mammary adipocyte diameter and aromatase expression [39,41,43]. Individual study Spearman correlation coefficients with 95% confidence intervals and pooled estimate using random-effects model. Diamond represents overall effect size. Considerable heterogeneity was observed (*I*^2^ = 82.9%). Pre = premenopausal; Post = postmenopausal.

Three independent studies (*n* = 333) [39,41,43] evaluated the correlation between mammary adipocyte diameter and aromatase expression. The pooled analysis demonstrated a moderate positive correlation (*r* = 0.36, 95% CI: 0.05–0.61, *p* = 0.0006) under a random-effects model (Figure 9), with substantial heterogeneity (*I*^2^ = 82.9%, *p* = 0.0006).

**Figure 10:**
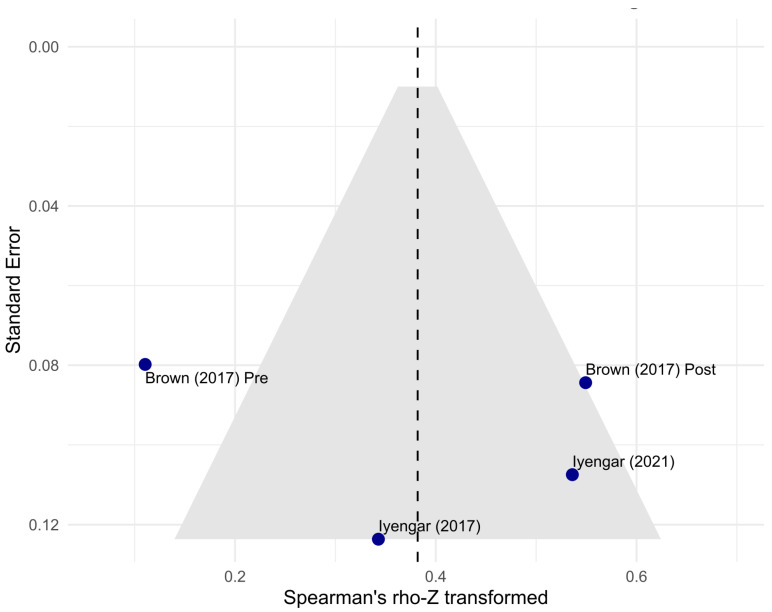
Funnel plot assessing publication bias in the correlation between mammary adipocyte diameter and aromatase expression [39,41,43]. Fisher’s Z-transformed correlation coefficients (with Bonett & Wright correction) plotted against standard error [25]. Each point represents one study. The vertical dashed line indicates the pooled Fisher’s Z-transformed correlation coefficient. The symmetrical distribution suggests no evidence of publication bias. Pre = premenopausal; Post = postmenopausal.

One study stratified results by menopausal status [43], revealing a significant correlation in postmenopausal women (*r* = 0.50, 95% CI: 0.37–0.61, *p* < 0.001) but not in premenopausal women (*r* = 0.11, 95% CI: −0.05–0.26, *p* = 0.290). The two remaining studies examined mixed populations (20% and 45% postmenopausal, respectively) and reported significant positive correlations (*r* = 0.33 and *r* = 0.49) [39,41] (Tables S2 and S3D). Leave-one-out sensitivity analysis identified the premenopausal subgroup [43] as the main source of heterogeneity (Figure S15). Excluding this subgroup markedly reduced heterogeneity (*I*^2^ = 82.9% to *I*^2^ = 3.4%) and increased the pooled correlation (*r* = 0.46, 95% CI: 0.24–0.64), supporting menopausal status as a key contributor to the observed heterogeneity (Figure S16). Funnel plot inspection showed symmetrical distribution (Figure 10), and Egger’s test did not detect publication bias (*t* = 0.36, *p* = 0.72). The trim-and-fill method identified no missing studies, further confirming the absence of publication bias (Figure S17).

##### Narrative Synthesis: Associations Between Mammary Adipocyte Diameter and Hormonal Biomarkers

Adipocyte diameter was significantly larger in women with elevated aromatase expression (CYP19A1 gene) compared to those with low expression (81.6 vs. 76.4 μm, *p* = 0.0156) [28].

Adipocyte diameter showed an inverse correlation with SHBG levels (*r* = −0.41) [41]. Higher HSD11B1 expression was associated with larger adipocytes (83.9 vs. 63.4 μm, *p* = 0.001), whereas HSD17B12 expression showed an opposite pattern (66.7 vs. 77.8 μm, *p* = 0.038) [28]. No associations were observed for HSD17B7 (*p* = 0.797) [28].

PPARG expression, a master regulator of adipocyte differentiation, likewise showed no association with adipocyte size (*p* = 0.683). These associations were observed independently of age, menopausal status, and experimental batch [28] (Table S3C).

### 3.7. Narrative Synthesis: Associations Between Mammary Adipocyte Size and Breast Cancer Characteristics

Seven studies examined associations between mammary adipocyte size and breast cancer characteristics, showing positive associations with cancer risk (*n* = 1), antiproliferative response (*n* = 1), tumor grade and stage (*n* = 1), smaller adipocytes in invasive lobular carcinomas (*n* = 2), no associations with the cellular proliferation marker Ki-67 (*n* = 1), hormone receptors, HER2, or estrogen receptor 1 gene (ESR1) (*n* = 1), and inconsistent tissue comparison results (*n* = 4).

Larger adipocytes were associated with a higher risk of ipsilateral invasive breast cancer (odds ratio (OR) = 1.25 per 10 μm, 95% CI: 1.02–1.54, *p* = 0.030) [40]. In another study, a greater adipocyte diameter was associated with an enhanced antiproliferative response (OR = 2.24, 95% CI:1.01–14.32, *p* = 0.046), while no association was observed with residual Ki-67 expression (diameter: OR = 1.52, 95% CI: 0.87–3.19, *p* = 0.148; area: OR = 1.08, 95% CI: 0.98–1.23, *p* = 0.124) [34]. Adipocyte area was also positively associated with the antiproliferative response (OR = 1.17, 95% CI: 1.01–1.74, *p* = 0.029) [34].

Analyses of tumor characteristics showed significant associations between adipocyte size and tumor aggressiveness. Larger adipocytes were observed in grade 3 compared with grade 1 tumors (81.1 vs. 74.2 μm, *p* = 0.019) and in stage 3 compared with stage 0 tumors (81.4 vs. 74.7 μm, *p* = 0.046) [28]. No association was found with hormone receptor status (ER *p* = 0.846; PR *p* = 0.749) or HER2 expression (*p* = 0.059) [28], or ESR1 expression (*p* = 0.133) [28]. Invasive lobular carcinomas (ILC) also tended to have smaller adipocytes than invasive carcinomas of no special type (NST) (β = −0.60, *p* = 0.07) [34]. Similarly, adipocytes in ILC were smaller compared with those in invasive mixed carcinomas (IMC: 75.1 ± 22.4 µm; ILC: 61.6 ± 22.6 µm, *p* < 0.001) [44].

Results from tissue comparisons were inconsistent. One study reported no difference in adipocyte area between tumor-adjacent and normal breast tissue (3798 vs. 3830 μm^2^, *p* = 0.900) [45], while another found smaller adipocytes in cancer-associated compared with normal breast tissue (2860 vs. 3398 μm^2^, *p* < 0.001) [44]. In population-based comparisons, cancer patients had smaller adipocytes than healthy controls (74.1 vs. 87.9 μm, *p* = 0.007, *n* = 22) [46]. In contrast, another study found larger adipocytes in benign breast disease (BBD)-cancer patients compared to volunteers and BBD-only patients (*p* < 0.001) [33] (Table S3D).

Table 1 summarizes the pooled effect estimates, heterogeneity, robustness, publication bias, and risk-of-bias profile across all meta-analyzed outcomes.

## 4. Discussion

This systematic review and meta-analysis demonstrate moderate positive associations between mammary adipocyte size and BMI, crown-like structure density, and aromatase expression. Our pooled analyses also showed that adipocytes were significantly larger in tissue with CLS presence versus absence, providing epidemiological evidence supporting the biological relevance of mammary adipose tissue features in metabolic dysfunction and breast cancer pathophysiology.

The association between mammary adipocyte size and BMI shows that breast adipose tissue participates in systemic metabolic processes. Mammary adipocytes share regulatory mechanisms with other fat depots and respond to hormonal signals such as insulin and leptin that regulate metabolism [47]. Beyond BMI, studies consistently showed positive associations with other adiposity indicators including waist circumference, waist-to-hip ratio, and DXA-measured truncal fat, suggesting that breast tissue functions similarly to central fat depots [48].

According to our meta-analysis, adipocyte size shows a positive association with crown-like structure density in mammary tissue. Furthermore, adipocytes were significantly larger in tissue with CLS presence compared to absence, confirming that hypertrophy precedes or accompanies inflammatory changes. These structures represent macrophage aggregation around adipocytes undergoing apoptosis [49]. As adipocytes enlarge, decreased surface area-to-volume ratios likely create oxygen and nutrient limitations leading to hypoxia [50]. However, evidence for adipose tissue hypoxia in humans remains inconsistent, with some studies reporting lower oxygen tension in obesity while others describe higher or depot-specific variations [51,52]. These conditions make enlarged adipocytes fragile and more susceptible to cell death, releasing fatty acids and debris that contribute to increased inflammatory responses [53]. Tissue-resident macrophages respond by undergoing M1 polarization, which is characterized by increased phagocytic activity and the secretion of pro-inflammatory mediators, including TNF-α, IL-6, and IL-1β [53]. These activated macrophages form crown-like structures around dying cells, creating inflammatory microenvironments that can affect epithelial cell behavior through paracrine signaling [14]. One study also reported positive associations between adipocyte size and IL-6 and leptin, with an inverse association with adiponectin [41], consistent with the positive association with hsCRP also reported independently in an earlier study [36], suggesting potential bidirectional relationships between local and systemic inflammation. From the methodological standpoint, most studies usually report macrophage number as a function of adipocyte number in each microscope field. The association observed between adipocyte size and macrophage infiltration is, in fact, opposite to what is expected when considering only the larger surface occupied by hypertrophic adipocytes on each microscope image.

These findings apply to metabolic dysfunction, with studies showing associations between larger adipocytes and metabolic markers, including diabetes, hypertension, insulin resistance, and lower SHBG levels. Progressive increases in adipocyte size with accumulating metabolic syndrome criteria were also reported. Enlarged adipocytes show impaired insulin sensitivity and increased fatty acid release [18,54]. The inverse correlation with SHBG is particularly relevant as lower levels increase bioavailable estrogens and androgens, creating a more estrogenic breast tissue environment that may partly explain how adipocyte hypertrophy contributes to breast cancer risk independently of BMI [46]. Additionally, 11β-hydroxysteroid dehydrogenase type 1 expression amplifies local glucocorticoid activity, promoting adipocyte hypertrophy [55].

Regarding cancer implications, our meta-analysis showed a moderate positive correlation between adipocyte diameter and aromatase expression in the breast, though substantial heterogeneity was observed. Aromatase contributes to local estrogen production in postmenopausal women [56]. Menopausal status appears to be the main determinant of mammary adipocyte size, acting independently of other lifestyle or metabolic factors [57]. In contrast, lifestyle factors such as smoking and high dietary fat intake primarily influence overall adiposity rather than breast-specific fat depots [57]. Adipocyte hypertrophy induces hypoxia and cellular stress, activating pro-inflammatory pathways that release TNF-α and IL-6, which in turn stimulate aromatase expression and enhance local estrogen production [58]. This illustrates the direct involvement of mammary adipose tissue in breast cancer development [35,36]. Additionally, one study reported associations between larger adipocytes and higher tumor grade and stage, suggesting that adipocyte hypertrophy may contribute to aggressive breast cancers through secretion of growth-promoting factors like leptin and inflammatory cytokines [28].

From a clinical perspective, these findings suggest that mammary adipocyte size could serve as a marker of microenvironmental alteration and carcinogenic risk to complement outcome stratification beyond BMI alone. Women with metabolically unhealthy adipose tissue despite normal weight represent a population that is not captured by standard anthropometric measures [39]. The integration of adipocyte measurement into clinical practice appears feasible, as peritumoral adipose tissue is routinely collected during breast biopsies and surgical procedures [59]. Digital pathology tools now enable standardized quantification of adipocyte morphology from routine histological sections [31]. However, standardization of measurement protocols and validation in prospective cohorts are needed before clinical implementation. These adipocyte-based metrics could provide additional information on tumor microenvironment characteristics and inform personalized surveillance and treatment strategies in breast cancer management [60].

Despite these promising associations, several methodological limitations must be acknowledged. Most studies had high or some concerns regarding risk of bias, primarily due to inadequate adjustment for key confounders such as age and menopausal status, and selection bias related to limited availability of mammary adipose tissue. The studies primarily used correlational methods that provide only point estimates and may not capture temporal complexity [61]. Measurement methods for adipocyte size varied considerably between studies, introducing methodological heterogeneity. Substantial heterogeneity was observed in meta-analyses of BMI and aromatase associations, likely reflecting population and methodological differences. Most studies used cross-sectional designs, limiting causal interpretations.

In contrast, this systematic review demonstrates several methodological strengths, including adherence to Cochrane guidelines [20,62], comprehensive database searches, and predefined eligibility criteria with independent data extraction. The use of random-effects models, ROBINS-E tool assessment [24], and sensitivity analyses provided conservative pooled estimates. Our meta-analyses provide novel quantitative estimates previously unavailable, including pooled correlations between adipocyte size and BMI, CLS density, and aromatase expression, and the mean difference in adipocyte size between adipose tissues with CLS presence and absence. Publication bias assessments showed no evidence of bias for main analyses [27]. Future studies should prioritize prospective designs with standardized measurement protocols and appropriate confounding control.

Based on the measurement approaches identified across the included studies (Table S2A), digital image analysis software was used in most studies (*n* = 17), most commonly at 20× magnification, with adipocyte counts ranging from 18 to 250 cells per sample and reproducibility formally reported in only one study (ICC = 0.89) [28]. This variability may contribute to measurement inconsistency and highlights the need for greater standardization and clearer reporting of measurement conditions in future studies.

## 5. Conclusions

This systematic review and meta-analysis synthesizes evidence on mammary adipocyte size and breast cancer outcomes. Despite methodological heterogeneity across studies, the overall findings demonstrate that increased adipocyte size is associated with more aggressive breast cancer characteristics, adiposity, metabolic dysfunction, and chronic local and systemic inflammation. Mammary adipocyte size therefore emerges as a promising morphological marker of adipose tissue dysfunction within the breast microenvironment, and further investigation in longitudinal and mechanistic studies is needed before its clinical utility can be established. Future studies should employ rigorous epidemiological approaches, including standardized assessment protocols, appropriate control for confounding factors, and prospective designs, to strengthen causal inference.

## Figures and Tables

**Table 1:** Summary of effect estimates and evidence credibility assessments across meta-analyzed outcomes.

Outcome	k (Studies); *n*	Pooled Estimate (95% CI)	Overall Heterogeneity *I*^2^	Robustness (Leave-One-Out)	Publication Bias (Egger’s *p*)	Risk of Bias (Contributing Studies)
BMI *	8; 720	0.44 [0.29 to 0.56]	72.8%	0.42 to 0.49	0.305	5 high; 3 some concerns
CLS density *	5; 383	0.45 [0.36 to 0.54]	0%	0.44 to 0.47	0.684	4 high; 1 some concerns
CLS status (+/−) ^†^	3; 297	9.69 µm [4.94 to 14.44]	0%	7.4 to 10.6 µm	0.354	3 high
Aromatase expression *	3; 333	0.36 [0.05 to 0.61]	82.9%	0.30 to 0.45	0.72	2 high; 1 some concerns

Estimates are pooled Spearman correlation coefficients unless otherwise noted. * Correlation coefficient (*r*); ^†^ Mean difference. k: number of contributing studies; *n*: total number of participants across contributing studies; BMI: body mass index; CLS: crown-like structures. Risk of bias assessed with the ROBINS-E tool (Figure 2); overall judgment per study (high/some concerns/low). No study contributing to these four outcomes was judged to be at low overall risk of bias.

## Data Availability

No new data were created in this study. All data analyzed were extracted from the published studies cited in the manuscript and are available in the article and its Supplementary Materials.

## Supplementary Materials

The following supporting information can be downloaded at: https://www.mdpi.com/article/10.3390/cancers18152464/s1. File S1: PRISMA 2020 checklist; Table S1: Search strategy: (A) Medline via PubMed; (B) Embase; (C) Web of Science; (D) CINAHL; (E) Cochrane Library; Table S2: (A) Summary of study characteristics across all eligible studies; (B) individual characteristics of included studies; Table S3: (A) Summary of studies examining the association between mammary adipocyte size and adiposity indicators; (B) summary of studies examining the association between mammary adipocyte size and inflammatory markers; (C) summary of studies examining the association between mammary adipocyte size and metabolic markers; (D) studies on mammary adipocyte size and breast cancer characteristics; Figure S1: Leave-one-out sensitivity analysis of the correlation between mammary adipocyte diameter and BMI [14,19,33,34,35,36,37,38]; Figure S2: Forest plot of the correlation between mammary adipocyte diameter and BMI [14,19,34,35,36,37,38] after excluding Shaik (2021) [33]; Figure S3: Sensitivity analysis comparing global estimates for the correlation between mammary adipocyte diameter and BMI before and after excluding Shaik (2021) [33]; Figure S4: Forest plot of the correlation between mammary adipocyte diameter and BMI [14,33,34,35,36,37] after excluding studies with estimated coefficients [19,38]; Figure S5: Sensitivity analysis comparing global estimates for the correlation between mammary adipocyte diameter and BMI before and after excluding studies with estimated coefficients [19,38]; Figure S6: Forest plot of the correlation between mammary adipocyte diameter and BMI [14,19,33,34,35,36,37,38], stratified by adipocyte measurement method (manual vs. digital/software-assisted); Figure S7: Forest plot of the correlation between mammary adipocyte diameter and BMI, stratified by adipocyte measurement method (manual vs. digital/software-assisted), after excluding Shaik (2021) [33]; Figure S8: Funnel plot with trim-and-fill method for the correlation between mammary adipocyte diameter and BMI; Figure S9: Leave-one-out sensitivity analysis of the correlation between mammary adipocyte diameter and CLS density [14,19,34,37,39]; Figure S10: Forest plot of the correlation between mammary adipocyte diameter and CLS density [34,37,39] after excluding studies with estimated coefficients [14,19]; Figure S11: Sensitivity analysis comparing global estimates for the correlation between mammary adipocyte diameter and CLS density before and after excluding studies with estimated coefficients [14,19]; Figure S12: Funnel plot with trim-and-fill method for the correlation between mammary adipocyte diameter and CLS density; Figure S13: Leave-one-out sensitivity analysis for mean difference in mammary adipocyte diameter between CLS-positive and CLS-negative mammary tissue [37,38,39]; Figure S14: Funnel plot with trim-and-fill method for mean difference in mammary adipocyte diameter between CLS-positive and CLS-negative mammary tissue; Figure S15: Leave-one-out sensitivity analysis for the correlation between mammary adipocyte diameter and aromatase expression [39,41,43]; Figure S16: Forest plot of the correlation between mammary adipocyte diameter and aromatase expression [39,41,43] after excluding the premenopausal subgroup [43]; Figure S17: Funnel plot with trim-and-fill method assessing publication bias in the correlation between mammary adipocyte diameter and aromatase expression.

## Abbreviations

The following abbreviations are used in this manuscript:

ALT	Alanine Aminotransferase
AST	Aspartate Aminotransferase
BBD	Benign Breast Disease
BMI	Body Mass Index
BWATi	Breast White Adipose Tissue Inflammation
CENTRAL	Cochrane Central Register of Controlled Trials
CI	Confidence Interval
CLS(s)	Crown-Like Structure(s)
CYP19A1	Cytochrome P450 family 19 subfamily A member 1
DXA	Dual-Energy X-Ray Absorptiometry
ER	Estrogen Receptor
ESR1	Estrogen Receptor 1 Gene
HER2	Human Epidermal Growth Factor Receptor 2
hsCRP	High-sensitivity C-Reactive Protein
HSD	Hydroxysteroid Dehydrogenase
ICC	Intraclass Correlation Coefficient
IGF-1	Insulin-like Growth Factor 1
IL-1β	Interleukin-1 Beta Protein
IL1B	Interleukin-1 Beta Gene
IL-6	Interleukin-6
ILC	Invasive Lobular Carcinoma
IMC	Invasive Mixed Carcinoma(s)
Ki-67	Ki-67 Cellular Proliferation Marker
LEP	Leptin Gene
MCP-1	Monocyte Chemoattractant Protein-1
MD	Mean Difference
MeSH	Medical Subject Headings
MetS	Metabolic Syndrome
NST	No Special Type
OR	Odds Ratio
PPARG	Peroxisome Proliferator-Activated Receptor Gamma
PR	Progesterone Receptor
PRISMA	Preferred Reporting Items for Systematic Reviews and Meta-Analyses
ROBINS-E	Risk of Bias in Non-randomized Studies of Exposure
SHBG	Sex Hormone-Binding Globulin
TAMs	Tumor-Associated Macrophages
TNF	Tumor Necrosis Factor
TNF-α	Tumor Necrosis Factor-Alpha
WHR	Waist-to-Hip Ratio
