# Supplementary material for: Mammary Adipocyte Size, Obesity-Related Breast Adipose Tissue Dysfunction, and Breast Cancer: A Systematic Review and Meta-Analysis
Source: Cancers (Basel). 2026 Jul 31;18(15):2464. doi: 10.3390/cancers18152464 (PMC13463851; doi:10.3390/cancers18152464)
Supplement: Supplementary file 1 [file cancers-18-02464-s001.zip › cancers-4432184-supplementary_30072026.pdf]

cancers

MDPI

Mammary adipocyte size, obesity-related breast adipose tissue dysfunction, and breast cancer: A systematic review and meta-analysis

Ouafa Badre, Sue-Ling Chang, Belkacem Abdous, Julie Lemieux, Francine Durocher, André Tchernof, Caroline Diorio

Supplementary Materials

Table S1.A. Search strategy for Medline via PubMed

Theme/Search Terms	Query	Line#	Results
Exposure [breast adipocyte size]	("breast*"[Title/Abstract] OR "breast tissue*"[Title/Abstract] OR "mammary tissue*"[Title/Abstract] OR "Human Mammary Glands"[Title/Abstract] OR "Mammary"[Title/Abstract] OR "mammary glands, human"[MeSH Terms]) AND ("Adipose Tissue"[Title/Abstract] OR "Adipocyte Number"[Title/Abstract] OR "Adipocyte Size"[Title/Abstract] OR "adipose tissue*"[Title/Abstract] OR "Adipose"[Title/Abstract] OR "adipocyte diameter*"[Title/Abstract] OR "Adipose Stroma"[Title/Abstract] OR "adipocyte hypertrophy"[Title/Abstract] OR "adipocyte*"[MeSH Terms] OR "Adipose Tissue"[MeSH Terms])	#1	4468
Outcomes I [Obesity and Metabolic Dysfunction]	"Metabolic Syndrome"[Title/Abstract] OR ("Metabolic"[Title/Abstract] AND "syndrome*"[Title/Abstract]) OR ("metabol*"[Title/Abstract] AND "syndrome*"[Title/Abstract]) OR "dyslipidemia"[Title/Abstract] OR "hypertens*"[Title/Abstract] OR "obese"[Title/Abstract] OR "Obesity"[Title/Abstract] OR "insulin resistant"[Title/Abstract] OR "insulin resistance"[Title/Abstract] OR (("type 2"[Title/Abstract] OR "type II"[Title/Abstract]) AND "diabetes"[Title/Abstract]) OR "type 2 Diabetes"[Title/Abstract] OR "inflammat*"[Title/Abstract] OR "cytokine"[Title/Abstract] OR "Metabolic Syndrome"[MeSH Terms] OR "Dyslipidemias"[MeSH Terms] OR "Hypertension"[MeSH Terms] OR "Obesity"[MeSH Terms] OR "Insulin Resistance"[MeSH Terms] OR	#2	2,105,866

Theme/Search Terms	Query	Line#	Results
	"Inflammation"[MeSH Terms] OR "cytokines"[MeSH Terms] OR "diabetes mellitus, type 2"[MeSH Terms]		
Outcomes II [Breast cancer]	(("breast*"[Title/Abstract] OR "breast tissue*"[Title/Abstract] OR "mammary tissue*"[Title/Abstract] OR "Human Mammary Glands"[Title/Abstract] OR "Mammary"[Title/Abstract]) OR("mammary glands, human"[MeSH Terms])) AND ("cancer*"[Title/Abstract] OR "carcinoma*"[Title/Abstract] OR "malignant neoplasm*"[Title/Abstract] OR "malignant tumor*"[Title/Abstract] OR "malignant tumour*"[Title/Abstract] OR "invasive cancer"[Title/Abstract] OR "carcinoma in situ"[Title/Abstract] OR "cancer cells"[Title/Abstract] OR "cancer risk"[Title/Abstract] OR "cancer survival"[Title/Abstract] OR "cancer features"[Title/Abstract] OR "tumor*"[Title/Abstract] OR "breast disease"[Title/Abstract])	#3	307,648
Total Outcomes	#2 OR #3	#4	2,385,434
	"Animals"[MeSH Terms] NOT "Humans"[MeSH Terms]	#5	1,792,347
Grouping (both objectives)	(#1 AND #4) NOT #5		2811

Table S1.B. Search strategy for Embase

Theme/Search Terms	Query	Line#	Results
Exposure [breast adipocyte size]	('breast*':ti,ab OR 'breast tissue*':ti,ab OR 'mammary tissue*':ti,ab OR 'human mammary glands':ti,ab OR 'mammary':ti,ab OR 'breast'/exp) AND ('adipose tissue':ti,ab OR 'adipocyte number':ti,ab OR 'adipocyte size':ti,ab OR 'adipose tissue*':ti,ab OR 'adipose':ti,ab OR 'adipocyte diameter*':ti,ab OR 'adipose stroma':ti,ab OR 'adipocyte hypertrophy':ti,ab OR 'adipocyte'/exp OR 'adipose tissue'/exp)	#1	10,224
Outcomes I [Obesity and Metabolic Dysfunction]	'metabolic syndrome x':ti,ab OR ('metabolic':ti,ab AND 'syndrome*':ti,ab) OR 'metabolic disorder':ti,ab OR ('metabol*':ti,ab AND 'syndrome*':ti,ab) OR 'dyslipidemia':ti,ab OR 'hypertens*':ti,ab OR 'obese':ti,ab OR 'obesity':ti,ab OR 'insulin resistant mellitus':ti,ab OR 'insulin resistance':ti,ab OR (('type 2':ti,ab OR 'type ii':ti,ab) AND 'diabetes':ti,ab) OR 'type 2 diabetes':ti,ab OR 'inflammat*':ti,ab OR 'cytokine':ti,ab OR 'metabolic syndrome x'/exp OR 'dyslipidemia'/exp OR 'hypertension'/exp OR 'obesity'/exp OR 'insulin resistance'/exp OR 'inflammation'/exp OR 'cytokine'/exp OR 'non insulin dependent diabetes mellitus'/exp	#2	5,691, 265
Outcomes II [Breast cancer]	('breast*':ti,ab OR 'breast tissue*':ti,ab OR 'mammary tissue*':ti,ab OR 'human mammary glands':ti,ab OR 'mammary':ti,ab OR 'breast'/exp) AND ('cancer*':ti,ab OR 'carcinoma*':ti,ab OR 'malignant neoplasm*':ti,ab OR 'malignant tumor*':ti,ab OR 'malignant tumour*':ti,ab OR 'invasive cancer':ti,ab OR 'carcinoma in situ':ti,ab OR 'cancer cells':ti,ab OR 'cancer risk':ti,ab OR 'cancer survival':ti,ab OR 'cancer features':ti,ab OR 'tumor*':ti,ab OR 'breast disease':ti,ab)	#3	503,394
Total Outcomes	#2 OR #3	#4	6,109, 181
[embase]/lim	[embase]/lim	#5	19,745, 260
Animal exclusion	'animal'/exp NOT 'human'/exp	#6	2,640, 057
Grouping (both objectives)	#1 AND #4 AND #5 NOT #6		6333

Table S1.C. Search strategy for Web of Science

Theme/Search Terms	Query	Line#	Results
Exposure [breast adipocyte size]	TS=("breast*" OR "breast tissue*" OR "mammary tissue*" OR "Human Mammary Glands" OR "Mammary") AND TS=("Adipose Tissue" OR "Adipocyte Number" OR "Adipocyte Size" OR "adipose tissue*" OR "Adipose" OR "adipocyte diameter*" OR "Adipose Stroma" OR "adipocyte hypertrophy")	#1	8968
Outcomes I [Obesity and Metabolic Dysfunction]	TS=("Metabolic Syndrome" OR ("Metabolic" AND "syndrome*") OR ("metabol*" AND "syndrome*") OR "dyslipidemia" OR "hypertens*" OR "obese" OR "Obesity" OR "insulin resistant" OR "insulin resistance" OR (("type 2" OR "type II") AND ("diabetes")) OR "type 2 Diabetes" OR "inflammat*" OR "cytokine")	#2	3,348,149
Outcomes II [Breast cancer]	TS=("breast*" OR "breast tissue*" OR "mammary tissue*" OR "Human Mammary Glands" OR "Mammary") AND TS=("cancer*" OR "carcinoma*" OR "malignant neoplasm*" OR "malignant tumor*" OR "malignant tumour*" OR "invasive cancer" OR "carcinoma in situ" OR "cancer cells" OR "cancer risk" OR "cancer survival" OR "cancer features" OR "tumor*" OR ("breast disease"))	#3	836,963
Total Outcomes	#2 OR #3	#4	4,132,662
Human Population	(men or women or patient or female or women or women or man or men or male or subjects or adults * or patient* or participant*) or population groups	#5	17,128,143
Exclusion animal	animal models OR Experimental animals or Laboratory animals or Research animals	#6	817,700
Combination exposure + outcomes	#1 AND #4	#7	6365
Inclusion human population	#7 AND #5	#8	3635
Grouping (both objectives)	#8 NOT #6		3707

Table S1.D. Search strategy for CINAHL

Theme/Search Terms	Query	Line#	Results
Exposure [breast adipocyte size]	(MH "Breast") OR (TI/AB: "breast" OR "breast tissue" OR "mammary tissue*" OR "Human Mammary Glands" OR "Mammary*")** AND (MH "Adipocytes") OR (MH "Adipose Tissue") OR (TI/AB: "Adipose Tissue" OR "Adipocyte Number" OR "Adipocyte Size" OR "adipose tissue*" OR "Adipose" OR "adipocyte diameter*" OR "Adipose Stroma" OR "adipocyte hypertrophy")	S1	1691
Outcomes I [Obesity and Metabolic Dysfunction]	(MH "Metabolic Syndrome X") OR (MH "Hyperlipidemia") OR (MH "Hypertension") OR (MH "Obesity") OR (MH "Insulin Resistance") OR (MH "Diabetes Mellitus, Type 2") OR (MH "Inflammation") OR (MH "Cytokines")<br>OR<br>(TI/AB: "Metabolic Syndrome" OR ("Metabolic" AND "syndrome*") OR ("metabol*" AND "syndrome*") OR "dyslipidemia" OR "hypertens*" OR "obese" OR "Obesity" OR "insulin resistant" OR "insulin resistance" OR (("type 2" OR "type II") AND ("diabetes*")) OR "type 2 Diabetes" OR "inflammat*" OR "cytokine")	S2	1,194,581
Outcomes II [Breast cancer]	(MH "Breast") OR (TI/AB: "breast" OR "breast tissue" OR "mammary tissue*" OR "Human Mammary Glands" OR "Mammary*")**<br>AND<br>(TI/AB: "cancer*" OR "carcinoma*" OR "malignant neoplasm*" OR "malignant tumor*" OR "malignant tumour*" OR "invasive cancer" OR "carcinoma in situ" OR "cancer cells" OR "cancer risk" OR "cancer survival" OR "cancer features" OR "tumor*" OR "breast disease")	S3	95,265
Total Outcomes	S2 OR S3	S4	1,262,158
Combinaison exposure + outcomes	S1 AND S4	S5	1064
Animal exclusion	(TI/AB: "animal model" OR "laboratory animal" OR "experimental animal*" OR "research animal*" OR "mice" OR "rat*" OR "murine")**	S6	4,961,084
Grouping (both objectives)	S5 NOT S6		659

Table S1.E. Search strategy for Cochrane Library

Theme/Search Terms	Query	Line#	Results
Exposure [breast adipocyte size]	((breast) OR (breast NEXT tissue) OR (mammary NEXT tissue) OR (human NEXT mammary NEXT glands) OR (mammary)):ti,ab,kw OR [mh "breast"] AND ((Adipose NEXT Tissue) OR (Adipocyte NEXT Number) OR (Adipocyte NEXT Size) OR (adipose NEXT tissue*) OR (Adipose) OR (adipocyte NEXT diameter*) OR (Adipose NEXT Stroma) OR (adipocyte NEXT hypertrophy)):ti,ab,kw OR [mh "Adipose Tissue"] OR [mh "Adipocytes"]	#1	347
Outcomes I [Obesity and Metabolic Dysfunction]	((metabolic syndrome) OR (dyslipidemia) OR (hypertens) OR (obese) OR (Obesity) OR (insulin NEXT resistant) OR (insulin NEXT resistance) OR (((type 2) OR (type II)) AND ((diabetes))) OR (type 2 Diabetes) OR (inflammat) OR (cytokine)):ti,ab,kw<br>OR<br>[mh "Metabolic Syndrome"] OR [mh "Dyslipidemias"] OR [mh "Hypertension"] OR [mh "Obesity"] OR [mh "Insulin Resistance"] OR [mh "Inflammation"] OR [mh "cytokines"] OR [mh "Diabetes Mellitus, Type 2"]	#2	205,461
Outcomes II [Breast cancer]	((breast) OR (breast NEXT tissue) OR (mammary NEXT tissue) OR (human NEXT mammary NEXT glands) OR (mammary)):ti,ab,kw OR [mh "breast"]<br>AND<br>((cancer*) OR (carcinoma*) OR (malignant NEXT neoplasm*) OR (malignant NEXT tumor*) OR (malignant NEXT tumour*) OR (invasive NEXT cancer) OR (carcinoma NEXT in NEXT situ) OR (cancer NEXT cells) OR (cancer NEXT risk) OR (cancer NEXT survival) OR (cancer NEXT features) OR (tumor*) OR (breast NEXT disease)):ti,ab,kw	#3	64,928
Total Outcomes	#2 OR #3	#4	266,809
Exclusion animal	-	#5	N/A
Grouping (both objectives)	#1 AND #4		355

Table S2.A: Summary of study characteristics across all eligible studies (*n* = 21)

Domain	Category	Details
**Study Design**	Type of study	Cross-sectional (*n* = 12), Retrospective (*n* = 4), Prospective observational (*n* = 2), Nested case-control (*n* = 2), Self-controlled paired (*n* = 1)
	Population source	Hospital-based (*n* = 18), Population-based (*n* = 3)
	Sample size	Range: 10 to 347 participants
	Follow-up	No longitudinal follow-up in most studies; follow-up (*n* = 3)
**Population**	Age	Mean age range: 43.4–70.3 years across studies
	Menopausal status	Postmenopausal only (*n* = 2), Mixed pre/postmenopausal (*n* = 17), Not reported (*n* = 2)
	Geographic distribution	Europe (*n* = 6), North America (*n* = 13), Asia (*n* = 2), Mexico (*n* = 1)
**Exposure**	**Mammary adipocyte size**	
	Measurement method	Digital image analysis (HALO®, Adiposoft, ImageJ, Canvas, Fiji, Definiens, deep learning) (*n* = 17), Manual measurements (*n* = 4)
	Magnification used	20× (*n* = 10), 10× (*n* = 1), 40× (*n* = 1), 4× (*n* = 1), Not reported/other (*n* = 8)
	Number of adipocytes measuredper sample	Range: 18 to 250 cells; 30 cells most common threshold (*n* = 5)
	Measurement reproducibility reported	Reported (*n* = 1; ICC = 0.89)
	Type of adipocyte measure	Diameter (μm) measured (*n* = 18), Area (μm²) measured (*n* = 6), number (*n* = 2)
	Adipocyte size (mean/median)	Range: 64–113 μm
	Tissue source	Mammary adipose tissue from breast biopsies, mastectomy specimens, or surgical resections (*n* = 21): all patients mastectomy (*n* = 9), mixed patient groups (*n* = 5), procedure not reported (*n* = 5), all patients tumorectomy (*n* = 2)
**Outcomes**	**Obesity and metabolic dysfunction**	
	**Adiposity markers**	
	BMI classification	Mean BMI range: 22–37 kg/m² (*n* = 19), Not reported (*n* = 2)
	Waist circumference and waist-hip	Reported (*n* = 2)

Domain	Category	Details
	**Inflammatory markers**	
	Circulating inflammatory markers	hsCRP, IL-6, leptin levels measured (*n* = 9)
	Tissue inflammation markers	TAM, IL-6, MCP-1, TNF expression assessed (*n* = 12)
	**Metabolic markers**	
	Metabolic syndrome component	Insulin levels, diabetic status, hypertension, liver enzymes (ALT, AST), MetS criteria assessed (*n* = 4)
	**Other metabolic markers**	
	Aromatase (CYP19A1)	mRNA expression in mammary adipose tissue, measured by qRT-PCR (*n* = 4)
	Steroidogenic enzymes and transport protein	HSD11B1, HSD17B12, HSD17B7 expression (*n* = 1)
		Sex hormone-binding globulin levels (*n* = 1)
	**Breast cancer characteristics**	
	Type of breast cancer	Predominantly invasive carcinoma (IDC, ILC, mixed types) (*n* = 15)
	ER/PR/HER2 status	ER-positive: 63%–100%; PR-positive: 52%–89%; HER2-positive: 5%–29%
	Tumor size	Mostly T1 or T2 (≤5 cm)
	Tumor grade	Mostly Grade 2 or 3
	TNM stage	Early-stage (I/II) predominant; limited data

BMI: body mass index; *n*: number of studies reporting the information; CLS: crown-like structures; hsCRP: high-sensitivity C-reactive protein; ALT: Alanine aminotransferase; AST: Aspartate aminotransferase; CYP19A1, cytochrome P450 family 19 subfamily A member 1; ER, estrogen receptor; HER2: human epidermal growth factor receptor 2; ICC: intraclass correlation coefficient; IDC: invasive ductal carcinoma; IL-6: interleukin-6; ILC: invasive lobular carcinoma; MCP-1, monocyte chemoattractant protein-1; MetS, metabolic syndrome; PR: progesterone receptor; qRT-PCR: quantitative reverse transcription polymerase chain reaction; TNF: tumor necrosis factor; TNM: tumor, node, metastasis; TAM: Tumor-associated macrophages; WAT: white adipose tissue; WHR: waist-hip ratio; HSD11B1/HSD17B12/HSD17B7: hydroxysteroid dehydrogenase types 1, 12, and 7; SHBG: sex hormone-binding globulin.

Table S2.B: Individual characteristics of included studies (*n* = 21)

Author et al., year	Country	Sample size	Study design	Age (years)	Menopausal status	Adipocyte diameter (μm)	Adipocyte area (μm²)	BMI (kg/m²)	Breast cancer type	Receptor status
Isnaldi et al., 2022 [34]	Belgium	56	Retrospective observational	68.0[M]	100% Post	76.1[m] (70.7–83.9)	NR	29.4[M]	NST (67.9%), ILC (32.1%)	ER+ 100%, PR+ 82.2%, HER2- 98.2%
Isnaldi et al., 2020 [31]	Belgium	10	Retrospective observational	70.3[M]	100% Post	Distant: 77.3[M], CAA: 50.0[M]	NR	28.4[M]	NST (50%), ILC (50%)	ER+ 100%, PR/HER2 NR
Vaysse et al., 2017 [19]	Norway	107	Cross-sectional	55.2[M]	70.1% Post	64.3[M] ± 12.9	NR	24.9[M]	IDC (81.1%), ILC (13.2%)	ER+ 90.7%, PR+ 69.2%, HER2+ 5.6%
Maliniak et al., 2020 [32]	USA	342	Retrospective cohort	55.3[M]	63.9% Post	NR	NR	29.1[M]	Invasive (Stage I–III)	ER+ 79%, PR NR, HER2 NR
Iyengar et al., 2017 [39]	USA	72	Prospective	47–49[m]	20% Post	94.3[M] vs 103.6[Mb]	NR	21.9[m]	Benign (20%), in situ carcinoma (25%), invasive carcinoma (55%)	HR+ 63%/57%[b], HER2+ 26%/29%[b], TNBC 11%/14%[b]
Iyengar et al., 2015 [35]	USA	237	Prospective	48.0[m]	39.2% Post	92[M] ± 16 vs 107[M] ± 12[c]	NR	25.3[m]	NR	ER+ 81.3%, PR+ 71.7%, HER2 NR
Zhao et al., 2020 [37]	China	136	Retrospective	52.0[M]	47.1% Post	85.3[M] ± 9.4 vs 95.9[M] ± 9.1[c]	NR	23.9[M]	IDC	Luminal A 5.9%, Luminal B 57.4%, HER2+ 3.6%, TNBC 17.6%
Iyengar et al., 2018 [36]	Taiwan/ USA	347	Cross-sectional cohort	51[m] vs 48[md]	47% Post (Taiwan)	105.6[m] vs 102.1[md]	NR	23.2[m] vs 25.4[md]	Invasive and non-invasive carcinoma	Taiwan: HR+/HER2- 63%, HER2+ 21%, TNBC 7%

**Author et al., year**	**Country**	**Sample size**	**Study design**	**Age (years)**	**Menopausal status**	**Adipocyte diameter (μm)**	**Adipocyte area (μm^2)**	**BMI (kg/m^2)**	**Breast cancer type**	**Receptor status**
Morris et al., 2011 [14]	USA	30	Cross-sectional	49.9[M]	47% Post	NR	NR	24.5[M]	Ipsilateral breast cancer (7%), Contralateral breast cancer (47%), Carcinoma in situ/2 (40%), Other primary cancer independent of breast (7%)	NR
Greenlee et al., 2019 [38]	USA	91	Cross-sectional pilot	69.0[m]	79% Post	105.6[M] vs 99.3[Mc]	NR	29.9[M]	Stage 0–III (Hispanic/ Latina)	ER+ 86%, PR+ 70%, HER2+ 8%
Laforest et al., 2021 [28]	Canada	164	Cross-sectional	52.5[M]	50% Post	40–117	NR	27.0[M]	Unilateral breast cancer	ER+ 89.6%, PR+ 84.2%, HER2+ 11.0%
Laforest et al., 2020 [46]	Canada	23	Cross-sectional	55.0[M]	65% Post	74.1[m] vs 87.9[me]	NR	25.6[m]	Cases (*n*=17), Controls (*n*=6)	ER+/PR+ 76.5% (cases), HER2+ ~5%
Iyengar et al., 2021 [41]	USA	100	Cross-sectional cohort	49.0[m]	45% Post	102.5[m]	NR	26.1[m]	Non-invasive or benign (36%), invasive (64%)	HR+/HER2- 88%, HER2+ 11%, TNBC 1%
Shaik et al., 2021 [33]	USA	152	Nested case-control	53.5–54.8[M]	Majority Post	NR	NR	31.5–33.1[M]	Benign breast disease ± progression	NR
Blaszczak et al., 2021 [29]	USA	12	Self-controlled paired	45.0[m]	5/12 Post	NR	NR	32.2[M]	Ductal carcinoma (11 invasive, 1 DCIS)	ER+/PR+/HER2- (5), TNBC (5), ER+/HER2+ (1)

**Author et al., year**	**Country**	**Sample size**	**Study design**	**Age (years)**	**Menopausal status**	**Adipocyte diameter (µm)**	**Adipocyte area (µm²)**	**BMI (kg/m²)**	**Breast cancer type**	**Receptor status**
Almekinders et al., 2021 [40]	Netherlands	276	Nested case-control	NR	Age >55 = Post	86.0[m]	NR	NR	DCIS	HR+/HER2- 62.3%, HR+/HER2+ 15.7%, HR-/HER2+ 18.5%, HR-/HER2- 2.8%
Brown et al., 2017 [43]	USA	161	Cross-sectional	44.5[M] vs 55.0[Mf]	36.6% Post	101.1[m] vs 111.0[mf]	NR	25.7[M]	Cancer/prophylactic mastectomy	HR+ 69.8%, HER2+ 15.9%, TNBC 14.3%
Denis et al., 2017 [42]	USA	39	Cross-sectional	58.9[M] vs 57.1[Mg]	Majority Post	NR	4744[M] vs 3198[Mg]	36.5[M] vs 30.4[Mg]	Healthy breast tissue (no cancer)	Not applicable
Miran et al., 2020 [45]	France	49	Cross-sectional	67.0[M] vs 43.4[Mh]	74.4% Post (cases)	NR	3798[M]	27.8[M] vs 25.2[Mh]	Invasive breast carcinoma (43 cases)	ER+ 67.4%, PR+ 62.8%, HER2+ 4.6%
Suárez-nájera et al., 201 [44]	Mexico	20	Cross-sectional	NR	NR	79.9[M]	NR	NR	IDC, ILC, IMC	NR
Wolf et al., 2024 [30]	Germany	30	Cross-sectional	61.0[m] vs 65.0[mi]	NR	NR	Periphery: 5741[m] vs 6910[m], 2nd row: 2345[m] vs 3849[m], CAA: 1541[m] vs 2377[mi]	22.4[m] vs 29.3[mi]	IDC 100%	ER-/PR-/HER2- 100%

BMI: body mass index; CAA: cancer-adjacent adipose tissue; DCIS: ductal carcinoma in situ; ER: estrogen receptor; HER2: human epidermal growth factor receptor 2; HR: hormone receptor; IDC: invasive ductal carcinoma; ILC: invasive lobular carcinoma; IMC: invasive mixed carcinoma; NR: not reported; NST: no special type carcinoma; Post: postmenopausal; PR: progesterone receptor; Pre: premenopausal; TNBC: triple-negative breast cancer; Periphery = Distant (≥2cm from tumor); CAA: 1st row (Cancer-Associated Adipocytes, adjacent to tumor); 2nd row: Intermediate position.

Notes: [M] Mean; [m] Median; [b] Without WATi vs with WATi; [c] CLS-B- vs CLS-B+; [d] Taiwan vs USA; [e] Cases vs controls; [f] Premenopausal vs postmenopausal; [g] Non-diabetic vs type 2 diabetes; [h] Controls vs cases; [i] BMI<25 vs BMI≥25.

Table S3-A. Summary of studies examining the association between mammary adipocyte size and adiposity indicators

Author, Year	Country	Sample size	Adipocyte measure	Adiposity indicators	Statistical analysis	Main effect	95% CI	*P*-value	Comments
Greenlee et al., 2019 [38]	USA	89	Diameter (mean)	BMI	Spearman correlation	*r* = 0.327	[0.122, 0.505]	0.0017	Estimated by PlotDigitizer, CI via Fisher transformation with Bonett & Wright correction
Isnaldi et al., 2020 [31]	Belgium	10	Diameter (mean) - Distant adipocytes	BMI	Spearman correlation	*r* = 0.65	[0.145, 0.926]	0.049	Small sample CI via Fisher transformation
			Area (mean) – Distant adipocytes	BMI	Spearman correlation	*r* = 0.71	NR	0.027	Small sample
			Diameter (mean) - CAAs	BMI	Spearman correlation	*r* = 0.87	NR	0.002	
			Area (mean) - CAAs		Spearman correlation	*r* = 0.89	NR	0.001	
			Diameter (categorical) - Distant adipocytes	BMI categories (18.5-24.9/25.0-29.9/≥30.0 kg/m²)	Mann-Kendall test	τ = 0.59	NR	0.035	
			Area (categorical) - Distant adipocytes	BMI categories (18.5-24.9/25.0-29.9/≥30.0 kg/m²)	Mann-Kendall test	τ = 0.65	NR	0.021	
			Diameter	BMI categories	Mann-Kendall test	τ = 0.75	NR	0.007	

Author, Year	Country	Sample size	Adipocyte measure	Adiposity indicators	Statistical analysis	Main effect	95% CI	*P*-value	Comments
			(categorical) - CAAs	(18.5-24.9/25.0-29.9/≥30.0 kg/m²)					
Isnaldi et al., 2022 [34]	Belgium	39	Area (categorical) - CAAs	BMI categories (18.5-24.9/25.0-29.9/≥30.0 kg/m²)	Mann-Kendall test	τ = 0.80	NR	0.004	
			Diameter (mean)	BMI	Spearman correlation	*r* = 0.399	[0.082, 0.641]	0.011	Distant CI via Fisher transformation with Bonett & Wright correction
		33	Diameter (mean)	BMI	Spearman correlation	*r* = 0.352	NR	0.044	CAA
		39	Area (mean)	BMI	Spearman correlation	*r* = 0.421	[0.108, 0.658]	0.007	Distant CI via Fisher transformation with Bonett & Wright correction
		33	Area (mean)	BMI	Spearman correlation	*r* = 0.355	NR	0.042	CAA
		33	Diameter (mean)	BMI	Kendall's τ	τ = 0.304	[0.096, 0.489]	0.031	CAA
		39	Diameter (mean)	BMI	Kendall's τ	τ = 0.323	[0.116, 0.504]	0.011	Distant
		33	Area (mean)	BMI	Kendall's τ	τ = 0.287	[0.076, 0.473]	0.041	CAA
		39	Area (mean)	BMI	Kendall's τ	τ = 0.340	[0.134, 0.517]	0.007	Distant
Iyengar et al., 2015 [35]	USA	101	Diameter (mean)	BMI	Spearman correlation	*r* = 0.63	[0.481, 0.743]	<0.001	CI via Fisher transformation with Bonett & Wright correction

**Author, Year**	**Country**	**Sample size**	**Adipocyte measure**	**Adiposity indicators**	**Statistical analysis**	**Main effect**	**95% CI**	***P*-value**	**Comments**
Iyengar et al., 2018 [36]	Taiwan and USA	72	Diameter (mean)	BMI	Spearman correlation	*r* = 0.43	[0.210,0.608]	0.010	CI via Fisher transformation with Bonett & Wright correction
			Diameter (mean)	Body fat %	Spearman correlation	*r* = 0.52	[0.314, 0.679]	<0.01	CI via Fisher transformation with Bonett & Wright correction
Laforest et al., 2021 [28]	Canada	161	Diameter (mean)	BMI (kg/m²)	Mean comparison	<25: 71.7 μm 25-30 kg/m²: 81.7 μm ≥30: 88.0 μm	[69.3, 74.0]; [79.1, 84.2]; [84.8, 91.2]	<0.0001	Adjusted for age, menopause
			Diameter (mean)	BMI (continuous)	Linear regression	β = 1.21 μm per kg/m² (6.05 μm per 5 kg/m²	NR	< 0.0001	Adjusted for age, menopause
			Diameter (mean)	Waist circumference (cm)	Mean comparison	<80: 68.5 μm; 80-88 cm: 79.1 μm; ≥88: 86.3 μm	[65.9, 71.0]; [76.2, 82.0]; [84.2, 88.5]	<0.0001	Adjusted for age, menopause
			Diameter (mean)	WHR	Mean comparison	≤0.80: 71.1 μm; 0.80-0.85: 83.2 μm; ≥0.85: 86.1 μm	[68.7, 73.6]; [80.2, 86.1]; [83.2, 89.0]	<0.0001	Adjusted for age, menopause
			Diameter (mean)	Breast non-dense area (Q1–Q5)	Mean comparison	Q1: 67.2 μm; Q5: 88.5 μm	[63.8, 70.6]; [85.1, 91.9]	<0.0001	Adjusted for age, menopause
			Diameter (mean)	Non-dense area (continuous)	Linear regression	β = 0.10 μm per cm²	NR	< 0.0001	Adjusted for age, menopause

Author, Year	Country	Sample size	Adipocyte measure	Adiposity indicators	Statistical analysis	Main effect	95% CI	*P*-value	Comments
			Diameter (mean)	BMI at age 18	Mean comparison	<20: 77.4 μm; ≥25: 85.3 μm	[74.8, 80.0]; [78.1, 92.6]	0.0456	Adjusted for age, menopause
			Diameter (mean)	ΔBMI (quintiles)	Linear trend	NR	NR	<0.0001	Adjusted for age, menopause
			Diameter (mean)	WC (continuous)	Linear regression	β = 0.65 (+3.25 μm per 5 cm)	NR	<0.0001	Adjusted for age, menopause, and batch
			Diameter (mean)	Birth weight (pounds)	Mean comparison	<6: 83.3 μm; 6-8: 79.0 μm >8: 75.8 μm	[78.6, 88.3]; [76.8, 81.2]; [70.9, 80.7]	0.0349	Adjusted for age, menopause
Maliniak et al., 2020 [32]	USA	81/342	Number of adipocytes	BMI	Spearman correlation	*r* = -0.47	[-0.64, -0.29]	<0.001	
Morris et al., 2011 [14]		29	Diameter (mean)	BMI	Spearman correlation	*r* = 0.62	[0.334, 0.801]	<0.001	CI via Fisher transformation with Bonett & Wright correction
Shaik et al., 2021 [33]	USA	147/152	Diameter (mean)	BMI	Spearman correlation	*r* = 0.13	[-0.033, 0.287]	0.132	Estimated by PlotDigitizer, CI via Fisher transformation with Bonett & Wright correction AA women; adipocyte diameter measured in initial benign tissue before cancer development
			Diameter (mean)	BMI	Linear regression	NR	NR	0.0996	
Vaysse et al., 2017 [19]	Norway	107	Diameter (mean)	BMI	Spearman correlation	*r* = 0.487	[0.317, 0.626]	<0.001	CI via Fisher transformation with Bonett & Wright correction

Author, Year	Country	Sample size	Adipocyte measure	Adiposity indicators	Statistical analysis	Main effect	95% CI	*P*-value	Comments
			Diameter (mean)	BMI group (<25(*n*=62) vs >25(*n*=45))	Mean comparison	<25: 60.4 µm (±11,9); >25: 69.0 µm (±12,4)	NR	0.012	
			Number of adipocytes	BMI group (<25 (*n*=62) vs >25(*n*=45))	Mean comparison	<25: 341; >25: 257 adipocytes	NR	<0.001	
			Diameter (mean)	Waist-to-hip ratio	Linear regression	NR	NR	0.057	
			Diameter (mean)	DXA truncal fat %	Linear regression	NR	NR	< 0.001	
Zhao et al., 2020 [37]	China	136	Diameter (mean)	BMI	Spearman correlation	r = 0.496	[0.345, 0.617]	<0.001	CI via Fisher transformation with Bonett & Wright correction
			Diameter (mean)	BMI (WHO categories) <25 *n*=91 vs >25 *n*=45	Mean comparison	<25: 86.5 µm (±9.27); >25: 95.8 µm (±10.57)	NR	<0.001	
			Diameter (mean)	BMI (WGOC categories) <24 (*n*=85) vs ≥24 (*n*=51)	Mean comparison	<25: 85.7 µm; >25: 94.1 µm	NR	<0.001	WGOC = Working Group on Obesity in China
Wolf et al., 202 [30]	Germany	30	Area (median) - 1st row	BMI (< 25 (*n*=10) vs ≥ 25 (*n*=20)	Mean comparison (Mann-Whitney U test)	BMI<25: 1541 (1043-2034) µm²; BMI≥25: 2377 µm² (1902-3350)	NR	0.019	Tumor-adjacent adipocytes (first row)

**Author, Year**	**Country**	**Sample size**	**Adipocyte measure**	**Adiposity indicators**	**Statistical analysis**	**Main effect**	**95% CI**	***P*-value**	**Comments**
			Area (median) - 2nd row	BMI (< 25 (*n*=10) vs ≥ 25 (*n*=20)	Mean comparison (Mann-Whitney U test)	BMI<25: 2345 μm² (1921-3018) μm²; BMI≥25: 3849 μm² (2374-4623)	NR	0.037	Tumor-adjacent adipocytes (second row)
			Area (median) - Periphery	BMI (< 25 (*n*=10) vs ≥ 25 (*n*=20)	Mean comparison (Mann-Whitney U test)	BMI<25: 5741 μm² (3720-6724); BMI≥25: 6910 μm² (5263-8176)	NR	0.067	Peripheral adipocytes (≥2 cm from tumor)

BMI: Body Mass Index; WC: Waist Circumference; WHR: Waist-to-Hip Ratio; WHO: World Health Organization; WGOC: Working Group on Obesity in China; CAA: Cancer-Associated Adipocytes; Q1–Q5: Quintiles 1 to 5; NR: Not reported; *r*: Spearman correlation coefficient; τ: Kendall's tau; β: Regression coefficient; CI: Confidence interval; ΔBMI: Change in BMI; Digitized: Values extracted from published graphs using digital extraction tools. AA: African American; DXA: Dual-energy X-ray Absorptiometry.

Table S3-B. Summary of studies examining the association between mammary adipocyte size and inflammatory markers

Author, Year	Country	Sample size	Adipocyte measure	Inflammation measure	Statistical analysis	Main effect	95% CI	*P*-value	Comments
Almekinders et al., 2021 [40]	Netherlands	276	Size (area, 75th percentile)	CLS/10 cm^2	Spearman correlation	r = 0.47	[0.367,0.562]	< 0.001	CI via Fisher transformation with Bonett & Wright correction
Blaszczak et al., 2021 [29]	USA	12	Area (mean)	CLS-B/cm^2	Pearson correlation	r = 0.26 (affected breast); r = 0.65 (unaffected breast)	NR	0.497 (affected breast); 0.032 (unaffected breast)	Preliminary study and small sample
Greenlee et al., 2019 [38]	USA	89	Diameter (mean)	CLS (+/-)	Mean comparison	CLS^-: 99.3 μm (16.9) (*n*=49) range: (59.7–135.2) CLS^+: 105.6 μm (14.1) (*n*=40) range:(59.7–139.8)	NR	0.06	
				Severe BWATi	Mean comparison	Severe BWATi: 108.3 (16.6); None: 99.3 (16.9)	NA	0.04	BWATi severity cutoff: 0.27 CLS-B/cm^2
				Severe BWATi	Mean comparison	Mild: 103.2 (11.4); None 99.3 (16.9)	NA	0.31	
Isnaldi et al., 2022 [34]	Belgium	39/56	Diameter (mean)	CLS (+/-)	Univariable regression	β = 0.81	[0.13, 1.50]	0.021	

Author, Year	Country	Sample size	Adipocyte measure	Inflammation measure	Statistical analysis	Main effect	95% CI	*P*-value	Comments
			Area (mean)	CLS (+/-)	Univariable regression	β = 0.79	[0.11, 1.46]	0.023	
			Diameter (mean)	CLS (+/-)	Multivariable regression	β = 0.61	[-0.07, 1.29]	0.077	Adjusted for BMI
			Area (mean)	CLS (+/-)	Multivariable regression	β = 0.59	[-0.08, 1.26]	0.083	Adjusted for BMI
			Diameter (mean)	CLS-B/cm²	Spearman correlation	*r* = 0.48	[0.184,0.699]	0.004	Distant tissue CI via Fisher transformation with Bonett & Wright correction
			Area (mean)	CLS-B/cm²	Spearman correlation	*r* = 0.48	[0.194,0.691]	0.004	Distant tissue
			Diameter (mean)	TAM	Univariable regression	β = 0.71	[-0.09, 1.51]	0.078	
			Area (mean)	TAM	Univariable regression	β = 0.70	[-0.08, 1.49]	0.075	
			Diameter (mean)	TAM	Multivariable regression	β = 0.58	[-0.17, 1.33]	0.122	Adjusted for BMI
			Area (mean)	TAM	Multivariable regression	β = 0.58	[-0.16, 1.32]	0.118	Adjusted for BMI
Iyengar et al., 2015 [35]	USA	101/237	Diameter (mean)	CLS (+/-)	Mean comparison	CLS⁻: 92 μm (16); CLS⁺: 107 μm (12)	NR	<0.001	Group sizes not reported
Iyengar et al., 2017 [39]	USA	72	Diameter (mean)	CLS (+/-)	Mean comparison	CLS⁻: 94.3 μm (13.8), *n*=44; CLS⁺: 103.6 μm (13.1), *n*=28	NR	0.01	
			Diameter (mean)	CLS-B/cm²	Spearman correlation	*r* = 0.36	[0.130,0.546]	<0.01	

Author, Year	Country	Sample size	Adipocyte measure	Inflammation measure	Statistical analysis	Main effect	95% CI	*P*-value	Comments
Iyengar et al., 2018 [36]	USA	72	Diameter (median)	hsCRP	Spearman correlation	*r* = 0.50	[0.30,0.66]	<0.01	
Iyengar et al., 2021 [41]	USA	100	Diameter (mean)	Adiponectin	Spearman correlation	*r* = -0.33	NR	<0.001	Sensitivity analysis consistent
			Diameter (mean)	hsCRP	Spearman correlation	*r* = 0.47	NR	<0.001	Sensitivity analysis consistent
			Diameter (mean)	IL-6	Spearman correlation	*r* = 0.47	NR	<0.001	Sensitivity analysis consistent
			Diameter (mean)	Leptin	Spearman correlation	*r* = 0.56	NR	<0.001	Sensitivity analysis consistent
			Diameter (median)	BWATi (+/-)	Median comparison	BWATi$^+$: 105.8 µm; BWATi$^-$: 95.9 µm	NR	<0.001	
			Diameter (median)	BWATi (+/-)	Median comparison	BWATi$^+$: 95.8 µm; BWATi$^-$: 85.6 µm	NR	0.024	BMI <25 only
Laforest et al., 2021 [28]	Canada	73/164	Diameter (mean)	IL-6 expression	Mean comparison	High: 81.3 µm; Low: 73.2 µm	High [77.3,85.2]; Low [69.5,77.0]	0.0022	Adjusted for age, menopause, and batch
		73/164	Diameter (mean)	LEP expression	Mean comparison	High: 83.4 µm; Low: 73.1 µm	High [79.8, 87.0]; Low [69.6, 76.6]	0.0002	Adjusted for age, menopause, and batch
		64/164	Diameter (mean)	TNF expression	Mean comparison	High: 83.0 µm; Low: 73.6 µm	High [79.3, 86.7]; Low [69.9, 77.3]	0.0008	Adjusted for age, menopause, and batch

Author, Year	Country	Sample size	Adipocyte measure	Inflammation measure	Statistical analysis	Main effect	95% CI	*P*-value	Comments
		164/164	Diameter (mean)	M1 macrophage correlation	Multivariable regression	NR	NR	<0.001	Adjusted for age and menopausal status
		164/164	Diameter (mean)	Total Macrophage correlation	Multivariable regression	NR	NR	<0.001	Adjusted for age and menopausal status
		72/164	Diameter (mean)	PTGS2 expression	Mean comparison	High: 81.8 µm; Low: 75.1 µm	[78.0, 85.5]; [71.3, 78.9]	0.019	Adjusted for age, menopause, and batch
		21/164	Diameter (mean)	IL1B expression	Mean comparison	High: 68.2 µm; Low: 71.5 µm	[59.2, 77.2]; [63.0, 79.9]	0.631	Adjusted for age, menopause, and batch
Maliniak et al., 2020 [32]	USA	342	Number of adipocytes	CLS-B/cm²	Spearman correlation	r = -0.18	[-0.40, 0.07]	0.12	
Morris et al., 2011 [14]	USA	29	Diameter (mean)	CLS-B/cm²	Spearman correlation	r = 0.58	[0.245,0.7862]	0.001	Estimated by PlotDigitizer, CI via Fisher transformation with Bonett & Wright correction
Shaik et al., 2021 [33]	USA	150	Diameter (mean)	CLS (+/-)	Linear regression	NR	NR	<0.001	AA women: adipocyte diameter measured in initial benign tissue before cancer development
			Diameter (mean)	≥5 CLS-B	Linear regression	NR	NR	0.022	
		140	Diameter (mean)	IL	Linear regression	NR	NR	0.051	

Author, Year	Country	Sample size	Adipocyte measure	Inflammation measure	Statistical analysis	Main effect	95% CI	*P*-value	Comments
Vaysse et al., 2017 [19]	Norway	107	Diameter (mean)	CLS (+/-)	Linear regression	NR	NR	0.001	
			Diameter (mean)	CLS-B/cm²	Linear association	NR	NR	0.002	
			Diameter (mean)	CLS-B/cm²	Spearman correlation	$r = 0.42$	[0.240,0.572]	<0.001	CI via Fisher transformation with Bonett & Wright correction
Zhao et al., 2020 [37]	China	136	Diameter (mean)	CLS (+/-) CLS-: n=81 vs CLS+: n= 55	Mean comparison	CLS^-: 85.32 µm (9.44); CLS^+: 95.89 µm (9.09)	NR	<0.001	
			Diameter (mean)	CLS-B/cm²	Spearman correlation	$r = 0.496$	[0.354,0.616]	<0.001	

CLS: Crown-like structures; CLS-B: Crown-like structures in breast tissue; BWATi: Breast white adipose tissue inflammation; TAM: Tumor-associated macrophages; hsCRP: High-sensitivity C-reactive protein; IL-6: Interleukin-6; IL: infiltrating lymphocytes; TNF: Tumor necrosis factor; LEP: Leptin; NR: Not reported; NA: Not applicable; 95% CI: 95% confidence interval; r: Spearman/Pearson correlation coefficient; β: Regression coefficient; AA: African American; M1: Type 1 macrophages; PTGS2: Prostaglandin-endoperoxide synthase 2; IL1B: Interleukin-1 beta.

Table S3-C. Summary of studies examining the association between mammary adipocyte size and metabolic markers

Author, Year	Country	Sample size	Exposure	Outcome	Statistical analysis	Main effect	95% CI	*P*-value	Comments
Denis et al., 2017 [42]	USA	39	Area (median)	Diabetes	Mean comparison	ND: 3,198.2 μm² (*n*=11); T2D: 4,744.1 μm² (*n*=28)	NR	0.008	Estimated by PlotDigitizer Adipocyte area in diabetics vs non-diabetics
Greenlee et al., 2019 [38]	USA	89	Diameter (mean)	ALT	Linear regression	NR	NR	<0.050	ALT vs adipocyte diameter
			Diameter (mean)	AST	Linear regression	NR	NR	0.170	AST vs adipocyte diameter
Iyengar et al., 2021 [41]	USA	100	Diameter (mean)	Insulin	Spearman correlation	$r = 0.40$	NR	<0.001	Sensitivity analysis shows similar trend
			Diameter (mean)	SHBG	Spearman correlation	$r = -0.41$	NR	<0.001	Sensitivity analysis shows similar trend
			Diameter (mean)	Aromatase expression	Spearman correlation	$r = 0.49$	[0.31, 0.63]	<0.001	Aromatase expression vs adipocyte size. Sensitivity analysis shows a similar trend; CIs estimated via Fisher's Z transformation with Bonett & Wright (2000) correction

Author, Year	Country	Sample size	Exposure	Outcome	Statistical analysis	Main effect	95% CI	*P*-value	Comments
Laforest et al., 2021 [46]	Canada	164/164	Diameter (mean)	Number of MetS criteria (0-4)	Linear regression	0: 75.5 µm; 4: 90.2 µm	NR	0.004	Adjusted for age and menopause
		21/164	Diameter (mean)	HSD11B1 expression	Mean comparison	High: 83.9 µm; Low: 63.4 µm	[77.3, 90.5]; [56.4, 70.4]	0.001	Adjusted for age, menopause, and batch
		24/164	Diameter (mean)	HSD17B12 expression	Mean comparison	High: 66.7 µm; Low: 77.8 µm	[59.5, 73.9]; [70.6, 85.1]	0.038	Adjusted for age, menopause, and batch
		18/164	Diameter (mean)	HSD17B7 expression	Mean comparison	High: 71.4 µm; Low: 73.5 µm	[60.7, 82.1]; [62.8, 84.3]	0.797	Adjusted for age, menopause, and batch
		24/164	Diameter (mean)	PPARG expression	Mean comparison	High: 71.0 µm; Low: 73.5 µm	[62.5, 79.5]; [65.0, 82.0]	0.683	Adjusted for age, menopause, and batch
		164/164	Diameter (mean)	Diabetic status	Mean comparison	Yes: 86.3 µm; No: 78.5 µm	[78.1, 94.6]; [76.7, 80.4]	0.070	Adjusted for age and menopausal status
		164/164	Diameter (mean)	Hypertension status	Mean comparison	Yes: 85.3 µm; No: 77.1 µm	[81.3, 89.3]; [75.1, 79.1]	0.001	Adjusted for age and menopausal status
		140/164	Diameter (mean)	Aromatase expression	Mean comparison	High: 81.6 µm; Low: 76.4 µm	[78.7, 84.4]; [73.6, 79.2]	0.016	Adjusted for age, menopause, and batch

Author, Year	Country	Sample size	Exposure	Outcome	Statistical analysis	Main effect	95% CI	*P*-value	Comments
Brown et al., 2017 [43]	USA	59/161	Diameter (mean)	Aromatase expression (postmenopausal)	Spearman correlation	$r = 0.50$	[0.37,0.61]	<0.001	CIs estimated via Fisher's Z transformation with Bonett & Wright (2000) correction
		102/161	Diameter (mean)	Aromatase expression (premenopausal)	Spearman correlation	$r = 0.11$	[-0.045, 0.26]	0.290	
Iyengar et al., 2017 [39]	USA	72	Diameter (mean)	Aromatase expression	Spearman correlation	$r = 0.33$	[0.10, 0.52]	<0.010	CIs estimated via Fisher's Z transformation with Bonett & Wright (2000) correction

ND: Non-diabetic; T2D: Type 2 Diabetes; ALT: Alanine aminotransferase; AST: Aspartate aminotransferase; SHBG: Sex Hormone-Binding Globulin; MetS: Metabolic Syndrome; HSD11B1: 11β-Hydroxysteroid dehydrogenase type 1; *r*: Spearman correlation coefficient; NR: Not reported; HSD17B12: 17β-Hydroxysteroid dehydrogenase type 12; HSD17B7: 17β-Hydroxysteroid dehydrogenase type 7; PPARG: Peroxisome proliferator-activated receptor gamma.

Table S3-D. Studies on mammary adipocyte size and breast cancer characteristics

Author, Year	Country	Sample size	Exposure	Outcome	Statistical analysis	Main effect	95% CI	*P*-value	Comments
Almekinders et al., 2021 [40]	Netherlands	276 (108 cases, 168 controls)	Adipocyte diameter 75th percentile	Ipsilateral invasive breast cancer	Logistic regression	OR = 1.25 per 10 μm	[1.02, 1.54]	0.030	DCIS cases vs controls, median follow-up 5.8 years
			Adipocyte area 75th percentile	Ipsilateral invasive breast cancer	Logistic regression	OR = 1.17 per 10^3 μm²	[1.01, 1.35]	0.039	Q 4 vs Q1: OR = 2.75 (1.25-6.05)
			Large adipocytes Q4 vs Q1-3	Ipsilateral invasive breast cancer	Conditional logistic regression	OR = 2.08	1.16-3.74	0.013	Q 4 vs Q1-3 combined
		52 (28 cases, 24 controls)	Large adipocytes Q4 + COX-2 high	Ipsilateral invasive breast cancer	Conditional logistic regression	OR = 12.0	3.10-46.3	<0.001	Small adipocytes Q1-3 + COX-2 low (5 cases, 26 controls)
Isnaldi et al., 2022 [34]	Belgium	56	Diameter (mean)	Antiproliferative response	Logistic regression	OR = 2.24	[1.01, 14.32]	0.046	
			Area (mean)	Antiproliferative response	Logistic regression	OR = 1.17	[1.01, 1.74]	0.029	
			Diameter (mean)	Residual Ki67	Logistic regression	OR = 1.52	[0.87, 3.19]	0.148	
			Area (mean)	Residual Ki67	Logistic regression	OR = 1.08	[0.98, 1.23]	0.124	
			Diameter (mean)	ILC vs NST	Linear regression	β = -0.60	[-1.27, -0.07]	0.077	Histological type
			Area (mean)	ILC vs NST	Linear regression	β = -0.61	[-1.28, -0.05]	0.071	Histological type
Laforest et al., 2020 [46]	Canada	22	Diameter (mean)	Cases vs controls	Mean comparison	Cases: 74.1 μm (66.1–80.3), *n*=6; Controls: 87.9	NA	0.007	Controls vs Cases

						(86.2–89.5) μm, *n*=16			
			Diameter (mean)	Menopausal status (overall)	Mean comparison	Postmenopausal adipocytes 9.7 μm larger	NR	0.051	Menopausal status
			Diameter (mean)	Menopausal status (cancer patients)	Mean comparison	Postmenopausal adipocytes larger	NR	0.055 (t-test); 0.048 (KS test)	Menopausal status
Laforest et al., 2021 [28]	Canada	164/164	Diameter (mean)	ER status	Mean comparison	ER+: 79.0 μm; ER−: 78.4 μm	[77.0, 80.9]; [72.4, 84.1]	0.846	Adjusted for age and menopause
		136/164	Diameter (mean)	HER2 status	Mean comparison	HER2+: 84.1 μm; HER2−: 78.3 μm	[78.4, 89.8]; [76.0, 80.5]	0.059	Adjusted for age and menopause
		164/164	Diameter (mean)	PR status	Mean comparison	PR+: 79.0 μm; PR−: 78.2 μm	[77.0, 81.0]; [73.5, 82.9]	0.749	Adjusted for age and menopause
		138/164	Diameter (mean)	Tumor grade	Mean comparison	Grade 1: 74.2 μm; Grade 2: 80.1 μm; Grade 3: 81.1 μm	[69.7, 78.7]; [77.4, 84.8]	0.019	Adjusted for age and menopause
		164/164	Diameter (mean)	Tumor size (mm)	Mean comparison	≤5mm: 78.8 μm; >50mm: 82.1 μm	[72.4, 79.9]; [75.8, 88.4]	0.194	Adjusted for age and menopause
		164/164	Diameter (mean)	Tumor stage (TNM)	Mean comparison	Stage 0: 74.7 μm; Stage 3: 81.4 μm	[67.9, 80.4]; [75.8, 87.0]	0.046	Adjusted for age and menopause
		140/164	Diameter (mean)	ESR1 expression	Mean comparison	High: 77.5 μm; Low: 80.5 μm	[74.8, 80.2]; [77.7, 83.2]	0.133	Adjusted for age, menopause, and batch
Shaik et al., 2021 [33]	USA	*n*=152 (50 Volunteer, 47 BBD only, 55 BBD-cancer)	Mean adipocyte diameter (μm)	Study cohort (3 groups: Volunteer/ BBD only/ BBD-cancer)	ANOVA (linear regression)	NR	NR	<0.001	AA women: adipocyte diameter measured in the initial benign tissue before

									cancer development
Miran et al., 2020 [45]	China	49	Area (mean)	Tumor-adjacent vs normal breast tissue	Mean comparison	AdipTa: 3798 μm²; AdipN: 3830 μm²	NR	0.900	-
Suárez-Nájera et al., 2017 [44]	Mexico	20 (NBTA: *n*=5; AABC: *n*=15)	Area (mean)	Breast cancer subtypes (NBTA vs AABC)	Mean comparison	NBTA: 3398 μm²; AABC: 2860 μm²	[3204, 3615]; [2792, 2925]	<0.001	CAAs within 100 μm of invasive front (AABC group)

ER: Estrogen Receptor; PR: Progesterone Receptor; HER2: Human Epidermal Growth Factor Receptor 2; ILC: Invasive Lobular Carcinoma; NST: No Special Type; DCIS: Ductal Carcinoma in Situ; NBTA: Normal breast tissue adipocytes; AABC: adipocytes associated to breast cancer; CAAs: Cancer-Associated Adipocytes; OR: Odds Ratio; β: Regression coefficient; *r*: Spearman correlation coefficient; NR: Not reported; KS: Kolmogorov-Smirnov test; CYP19A1: Cytochrome P450 19A1 (aromatase gene); AdipN: Normal adipose tissue sample; AdipTa: Tumor-adjacent adipose tissue sample; BBD: Benign breast disease; AA; African American; ESR1: Estrogen receptor 1; COX-2: Cyclooxygenase-2; TNM: Tumor, Node, Metastasis staging system; Q: Quartile.

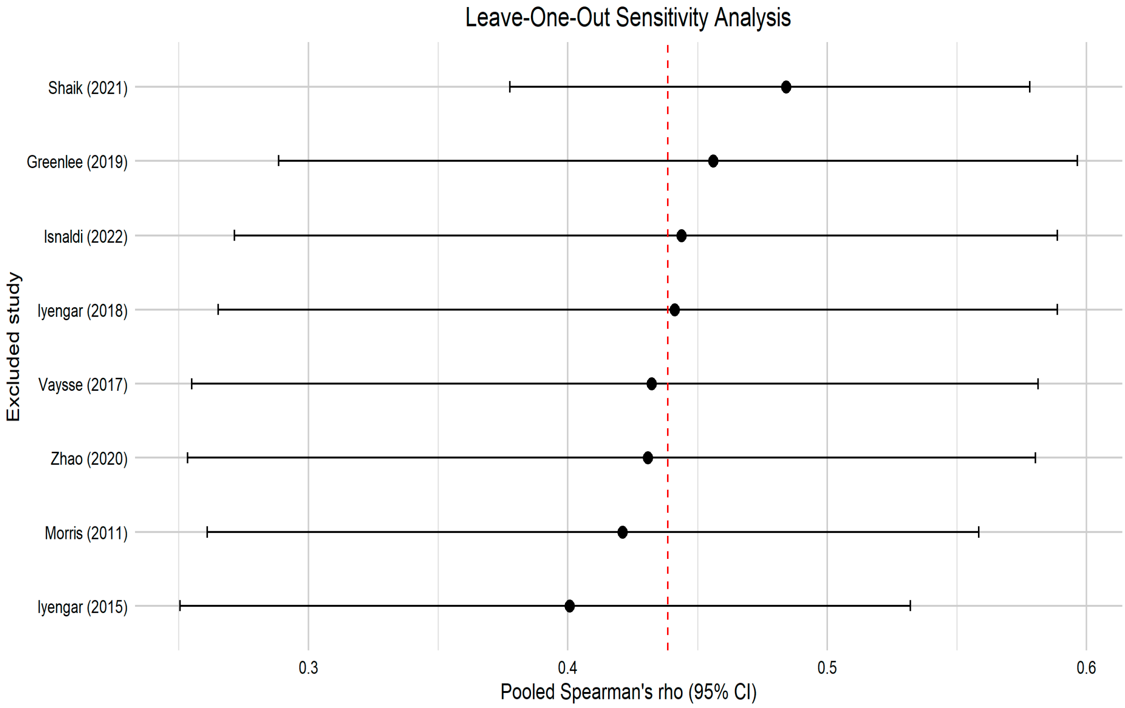

Figure S1. Leave-one-out sensitivity analysis of the correlation between mammary adipocyte diameter and BMI. Influence of each study on the overall Spearman correlation. Each line represents the pooled correlation coefficient and 95% confidence interval after excluding one study at a time [14,19,33–38]. The vertical dashed line represents the overall pooled estimate. Removing any single study did not substantially alter the overall estimate; however, excluding Shaik (2021) [33] markedly narrowed the confidence interval, suggesting it may be the main contributor to the observed heterogeneity. BMI = body mass index.

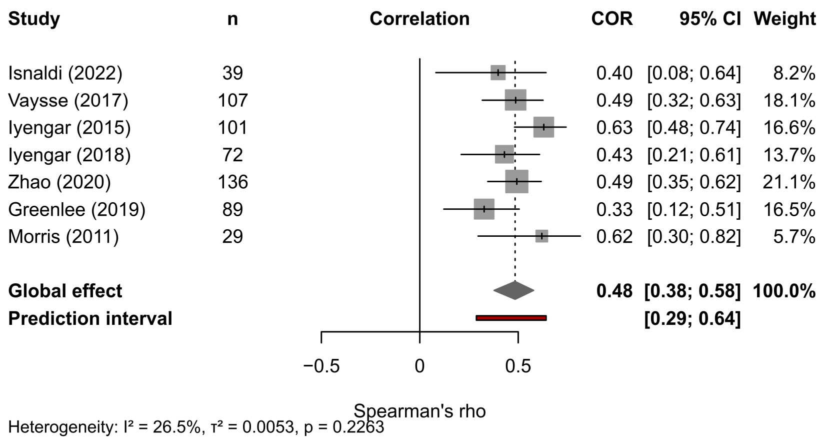

Figure S2. Forest plot of the correlation between mammary adipocyte diameter and BMI [14,19,34–38] after excluding Shaik (2021) [33]. Meta-analysis excluding the study with healthy tissue measurements. The diamond represents the overall effect size. Reduced heterogeneity was observed. BMI = body mass index.

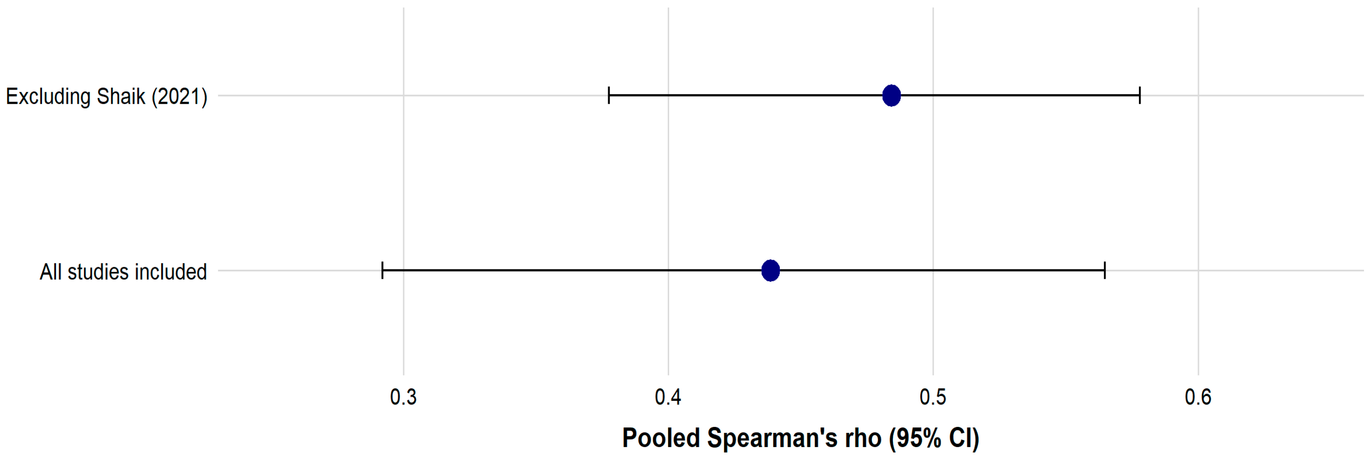

Figure S3. Sensitivity analysis: comparison of global estimates for the correlation between mammary adipocyte diameter and BMI. Comparison of pooled Spearman correlations including all studies versus excluding Shaik (2021) [33]. The analysis shows improved precision and slightly stronger correlation when excluding the study with healthy tissue measurements. BMI = body mass index.

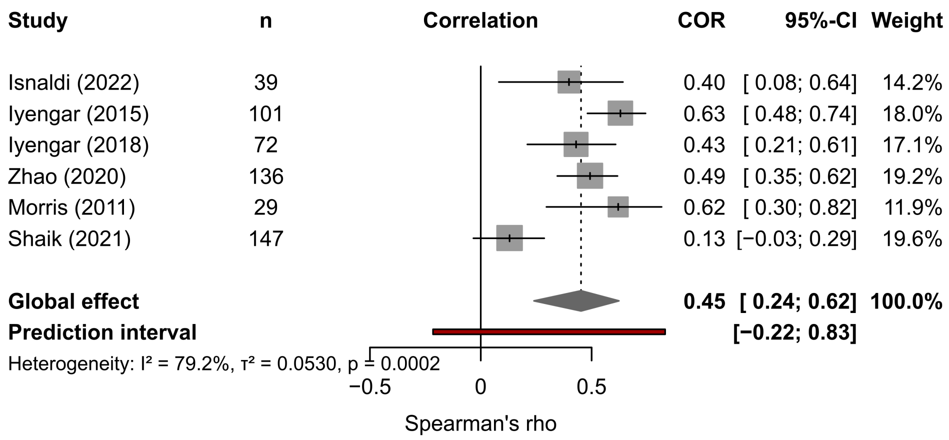

Figure S4. Forest plot of the correlation between mammary adipocyte diameter and BMI [14,33–37] after excluding studies with estimated coefficients. Meta-analysis excluding two studies [19,38] where correlation coefficients were estimated using digitized graphs. The diamond represents the overall effect size. Reduced heterogeneity was observed. BMI = body mass index.

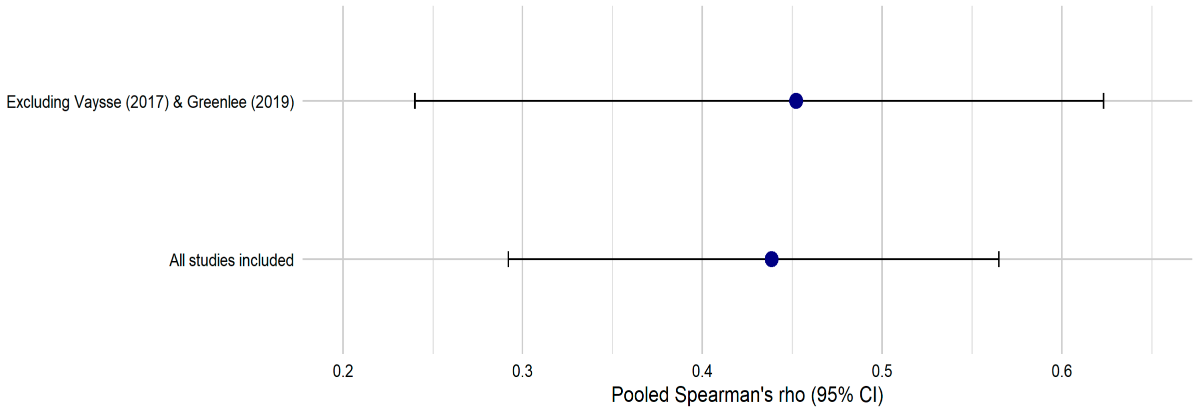

Figure S5. Sensitivity analysis comparing global estimates for the correlation between mammary adipocyte diameter and BMI before and after excluding studies with estimated coefficients. Comparison of the overall correlation coefficient when all studies are included versus when studies with reconstructed data are excluded [19,38]. The results remained consistent, further supporting the robustness of the findings. BMI = body mass index.

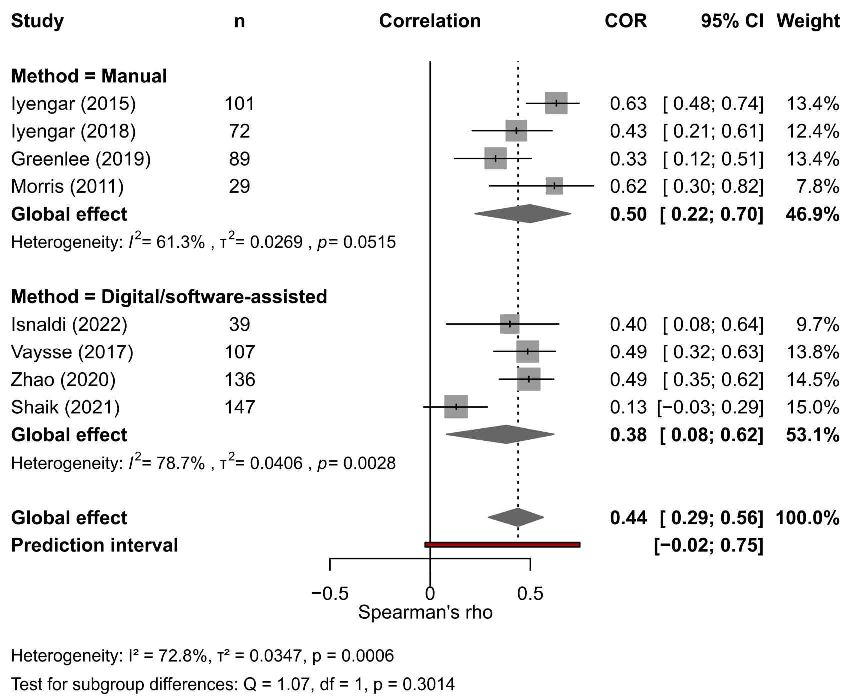

Figure S6. Forest plot of the correlation between mammary adipocyte diameter and BMI [14,19,33–38], stratified by adipocyte measurement method (manual vs. digital/software-assisted). Individual study Spearman correlation coefficients with 95% confidence intervals are shown within each subgroup, alongside subgroup and overall pooled estimates using a random-effects model. Substantial heterogeneity was observed in the digital/software-assisted subgroup, driven by Shaik (2021) [33]. BMI = body mass index.

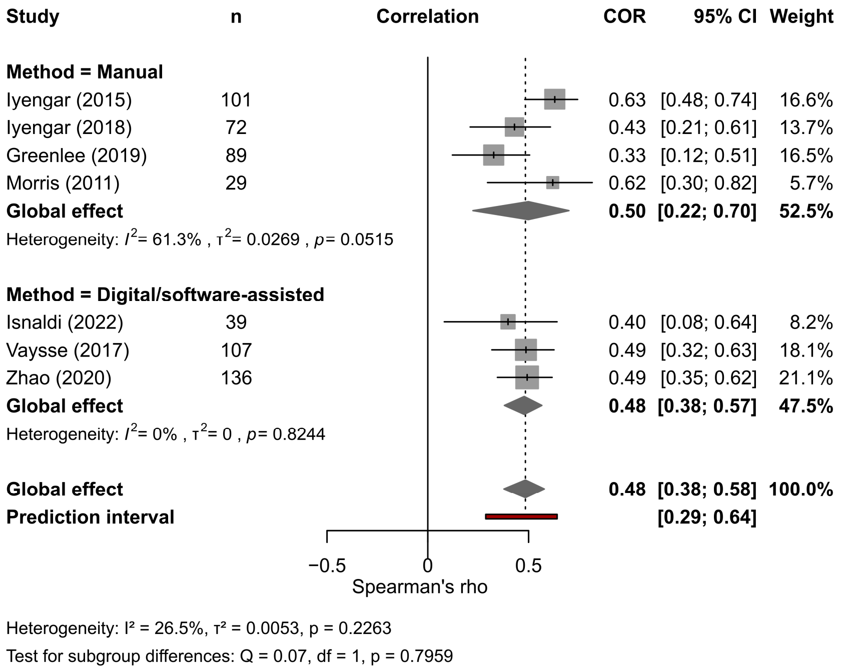

Figure S7. Forest plot of the correlation between mammary adipocyte diameter and BMI [14,19,34–38], stratified by adipocyte measurement method (manual vs. digital/software-assisted), after excluding Shaik (2021) [33]. Meta-analysis excluding the study driving heterogeneity in the digital/software-assisted subgroup. The diamonds represent the pooled effect size for each subgroup and overall. Heterogeneity was eliminated in the digital/software-assisted subgroup, and the two subgroups became nearly identical. BMI = body mass index.

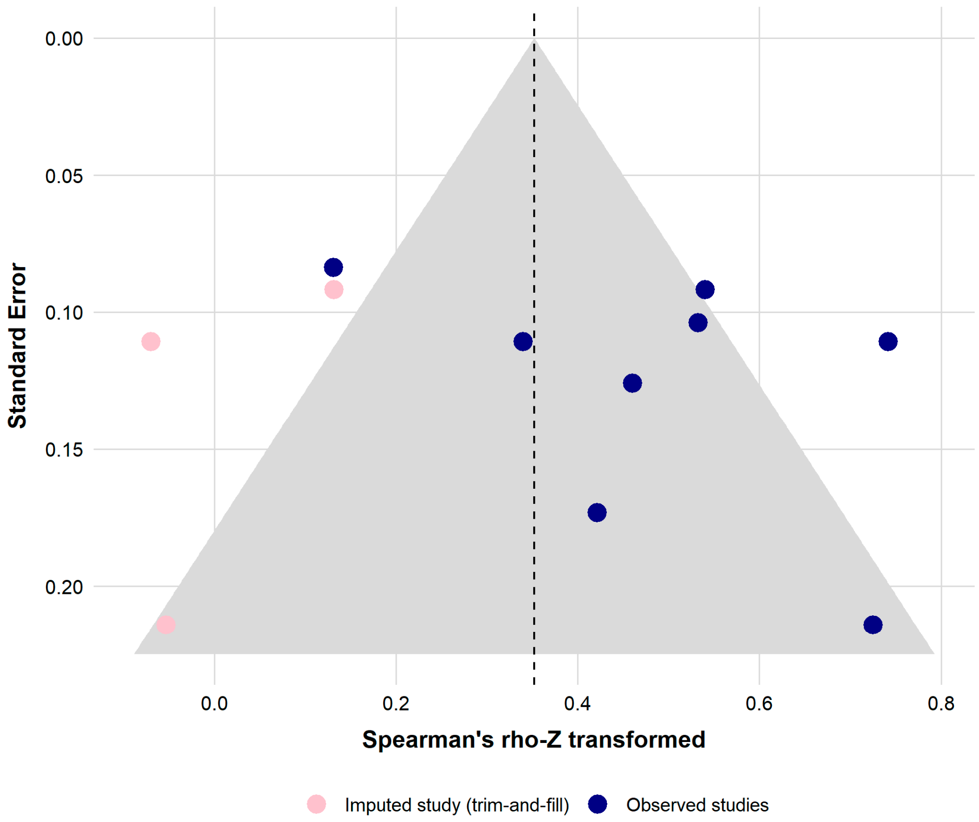

Figure S8. Funnel plot with trim-and-fill method for the correlation between mammary adipocyte diameter and BMI. Funnel plot with the application of the trim-and-fill method. Three studies were imputed on the left (lower correlation) side to correct for a slight asymmetry, yielding an attenuated but still positive adjusted estimate. Blue dots represent observed studies; pink dots represent imputed studies. BMI = body mass index.

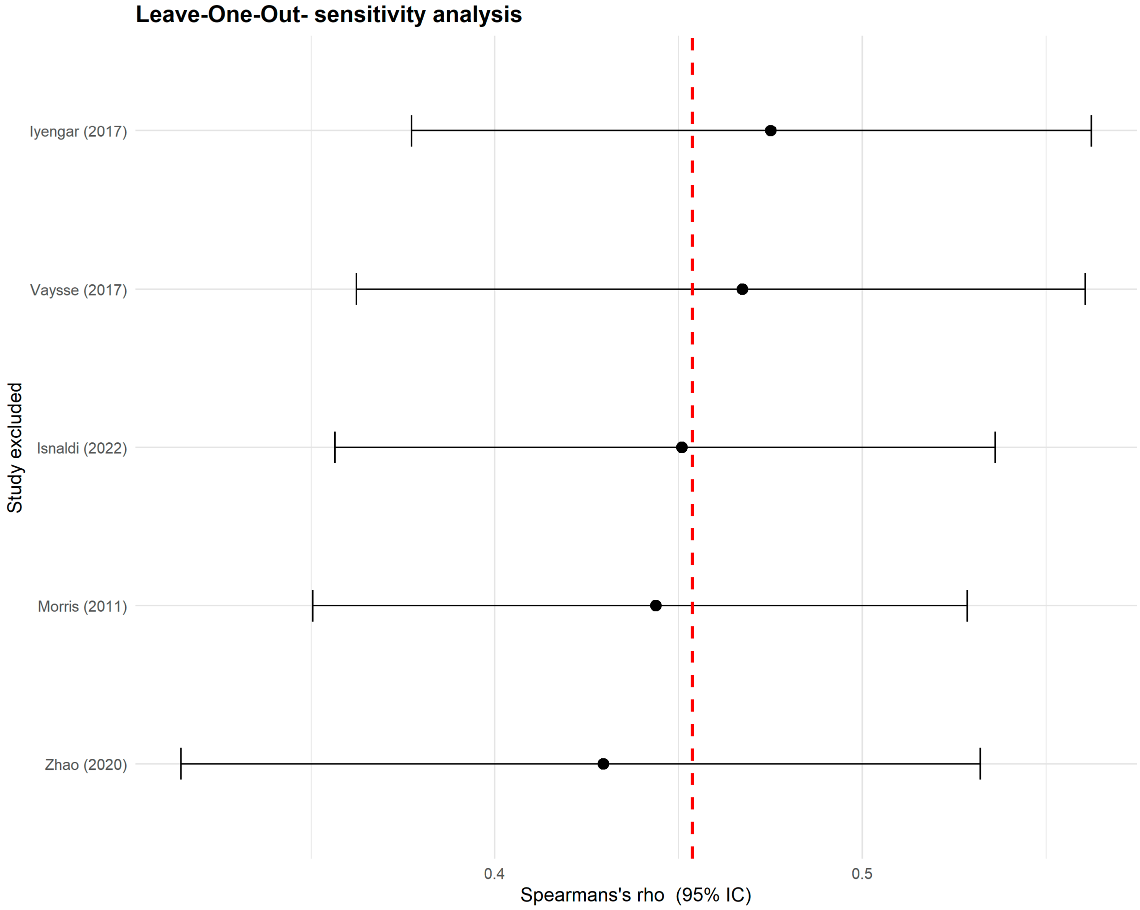

Figure S9. Leave-one-out sensitivity analysis of the correlation between mammary adipocyte diameter and CLS density [14,19,34,37,39]. Influence of each study on the overall pooled correlation coefficient. The vertical dashed line represents the overall pooled estimate. Removing any single study did not substantially alter the overall result, indicating the robustness of the meta-analytic estimate. CLS = crown-like structures.

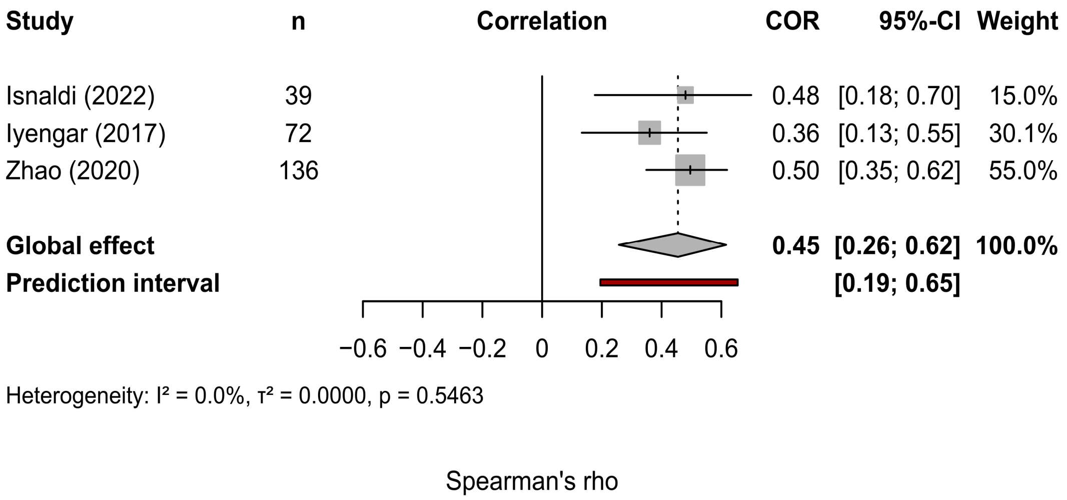

Figure S10. Forest plot of the correlation between mammary adipocyte diameter and CLS density [34,37,39] after excluding studies with estimated coefficients. Meta-analysis excluding two studies [14,19] where correlation coefficients were estimated using digitized graphs. The diamond represents the overall effect size. No heterogeneity was observed. CLS = crown-like structures.

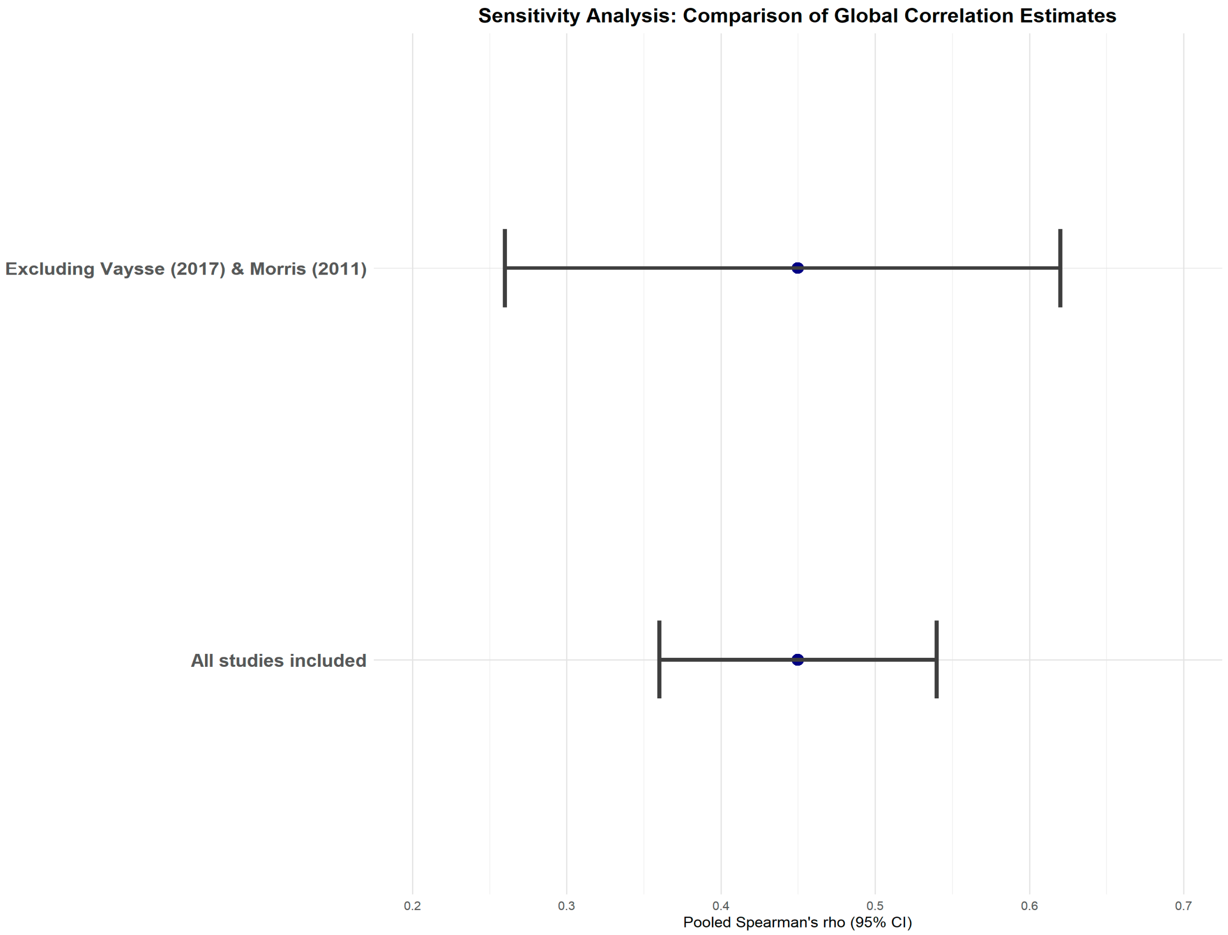

Figure S11. Sensitivity analysis comparing global estimates for the correlation between mammary adipocyte diameter and CLS density before and after excluding studies with estimated coefficients [14,19]. Comparison of the overall pooled correlation coefficient when all studies are included versus when studies with reconstructed data are excluded. The results remained consistent, further supporting the robustness of the findings. CLS = crown-like structures.

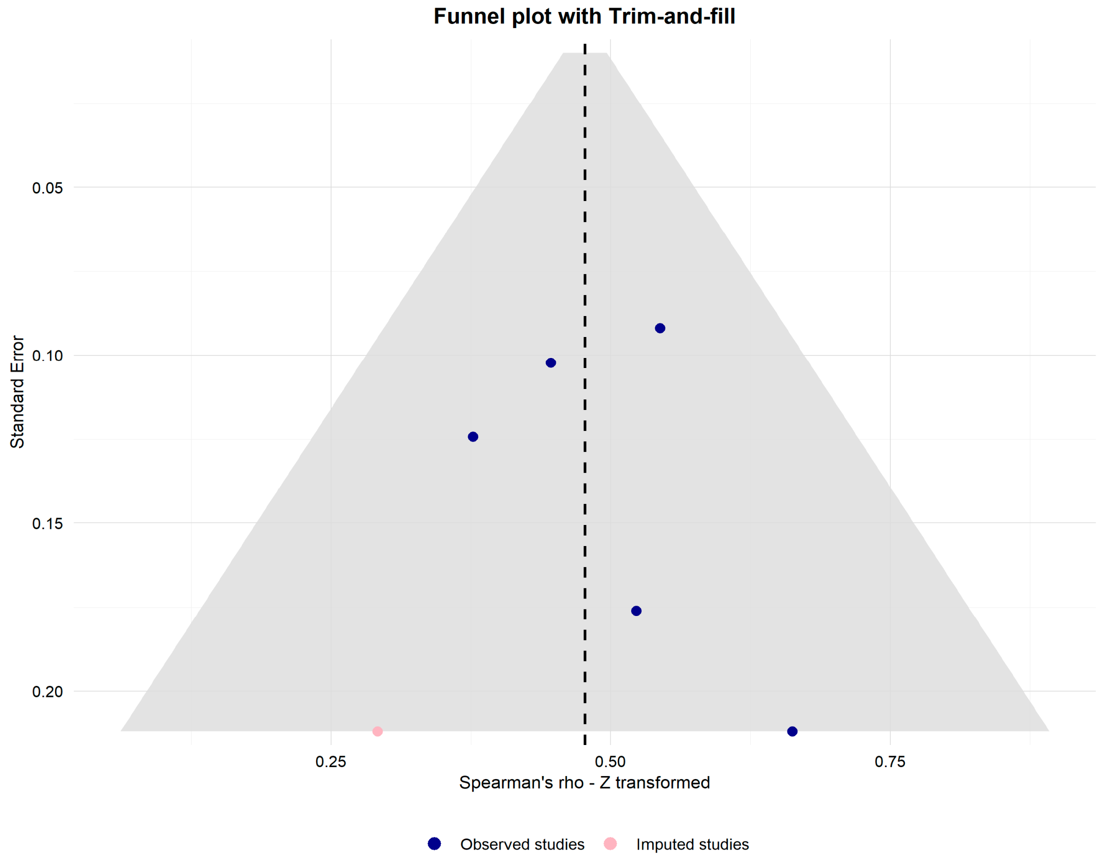

Figure S12. Funnel plot with trim-and-fill method for the correlation between mammary adipocyte diameter and CLS density. Funnel plot with the application of the trim-and-fill method. One imputed study was added, but the overall distribution remained symmetrical, indicating minimal risk of publication bias in the meta-analysis. The Duval and Tweedie trim-and-fill method was used to adjust for publication bias. Blue dots represent observed studies; the pink dot represents the imputed study. CLS = crown-like structures.

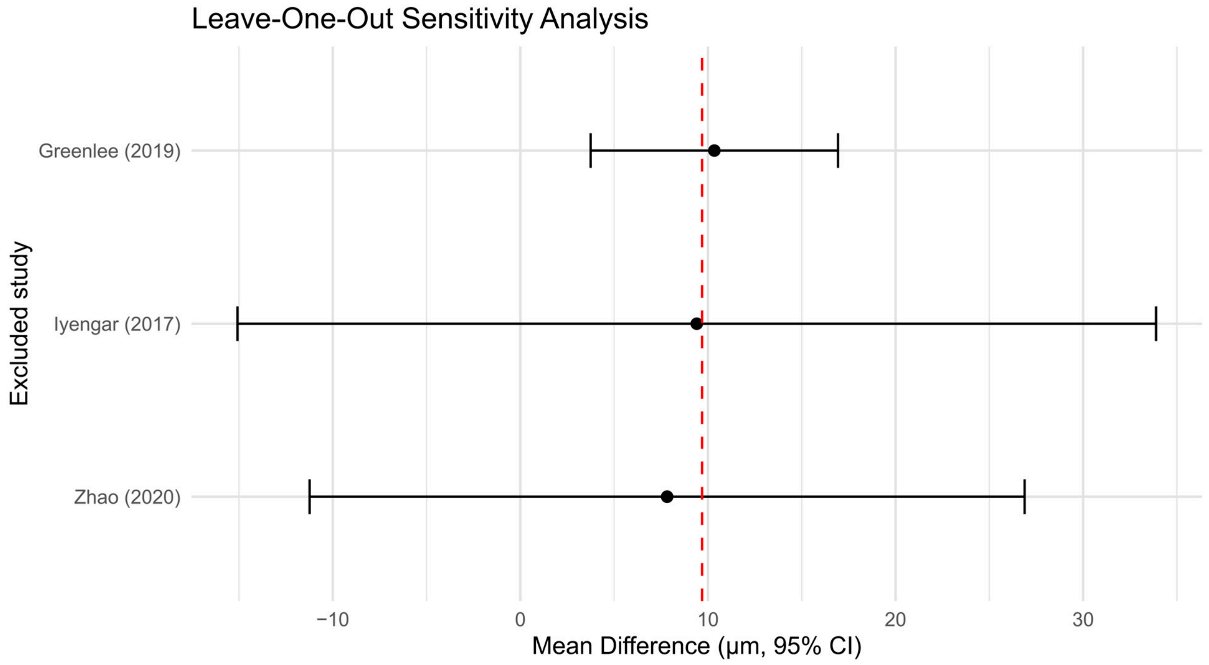

Figure S13. Leave-one-out sensitivity analysis for mean difference in mammary adipocyte diameter between CLS-positive and CLS-negative mammary tissue [37–39]. Each point represents the pooled mean difference when one study is excluded. The vertical dashed line represents the overall pooled estimate. The findings demonstrate robustness and stability of the results. MD = mean difference; CI = confidence interval; CLS = crown-like structures.

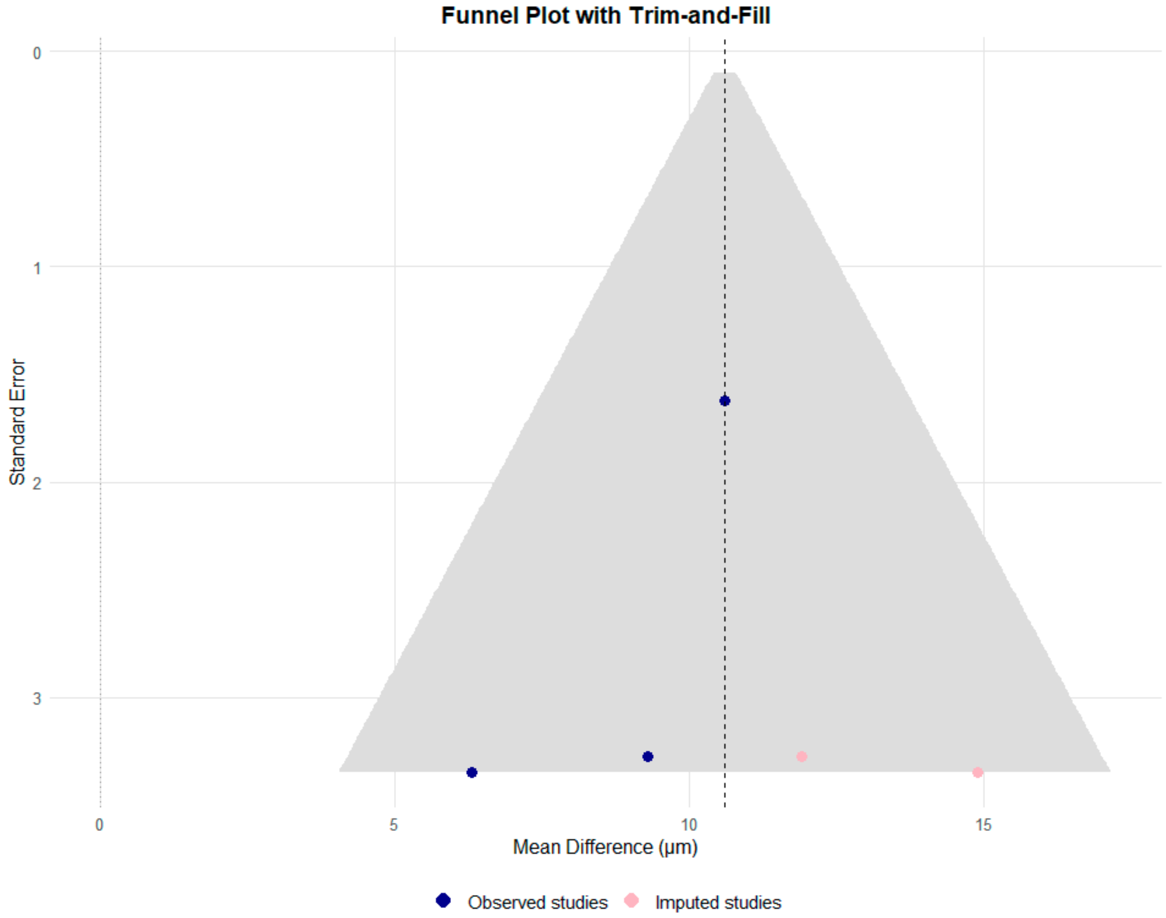

Figure S14. Funnel plot with trim-and-fill method for mean difference in mammary adipocyte diameter between CLS-positive and CLS-negative mammary tissue. Funnel plot with the application of the trim-and-fill method. Two imputed studies were added, but the overall distribution remained symmetrical, indicating minimal risk of publication bias in the meta-analysis. The Duval and Tweedie trim-and-fill method was used to adjust for publication bias. Blue dots represent observed studies; pink dots represent the imputed studies. CLS = crown-like structures.

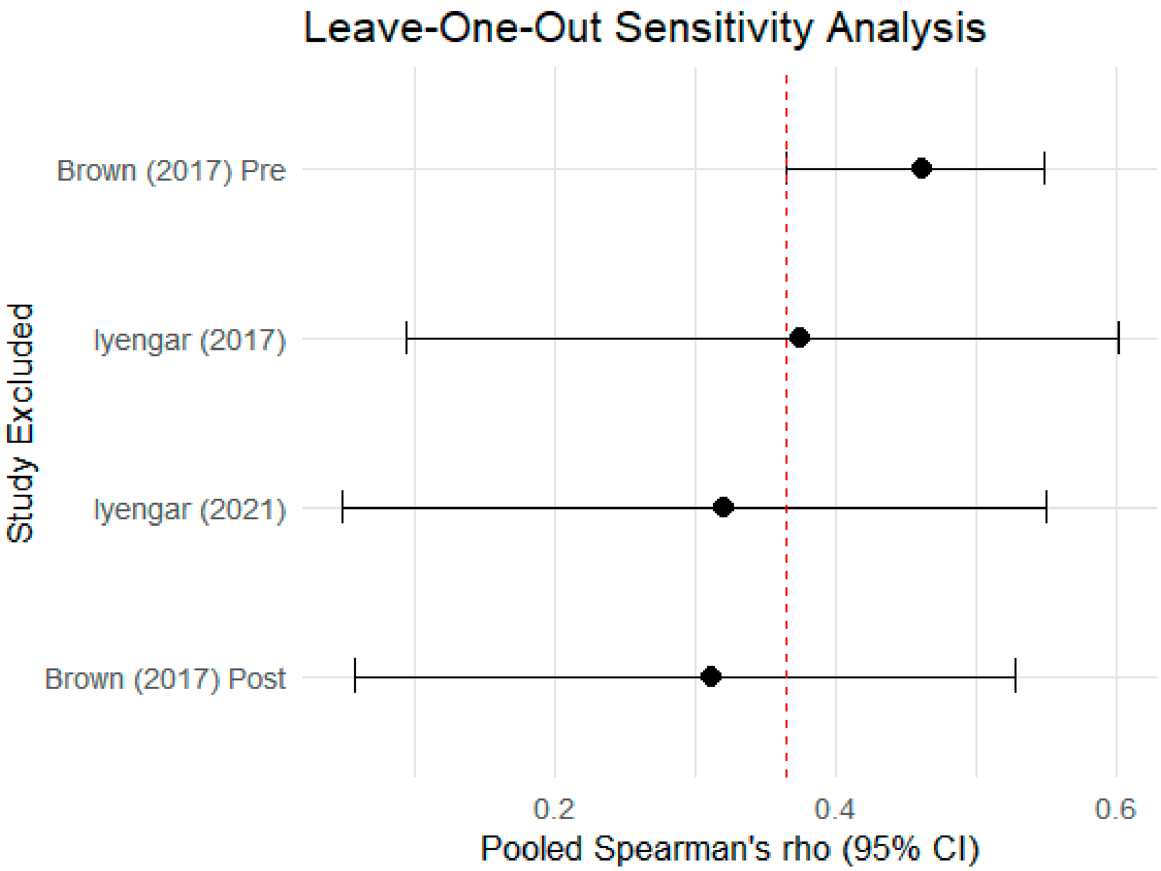

Figure S15. Leave-one-out sensitivity analysis for the correlation between mammary adipocyte diameter and aromatase expression [39,41,43]. Leave-one-out analysis showing the pooled Spearman correlation with 95% confidence intervals after sequential exclusion of each study. The vertical dashed line indicates the pooled estimate from all studies. Pre = premenopausal; Post = postmenopausal.

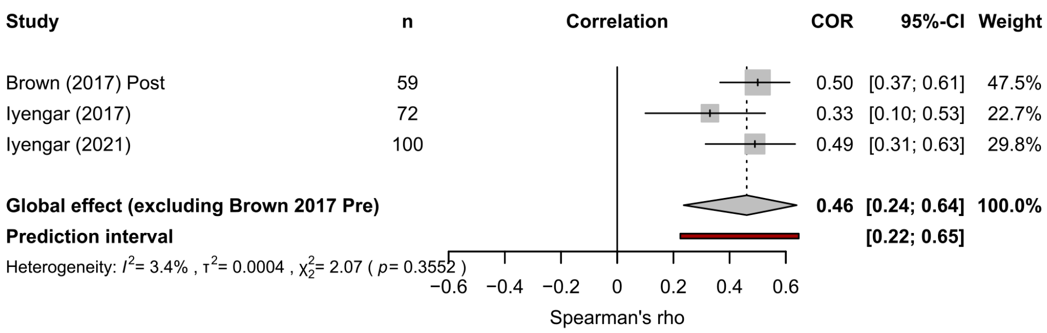

Figure S16. Forest plot of the correlation between mammary adipocyte diameter and aromatase expression [39,41,43] after excluding the premenopausal subgroup [43]. Meta-analysis excluding the subgroup responsible for most of the heterogeneity. The diamond represents the overall effect size. Heterogeneity was markedly reduced (I^2 = 3.4%), and the pooled correlation increased (r = 0.46), supporting menopausal status as a key contributor to the heterogeneity in this outcome.

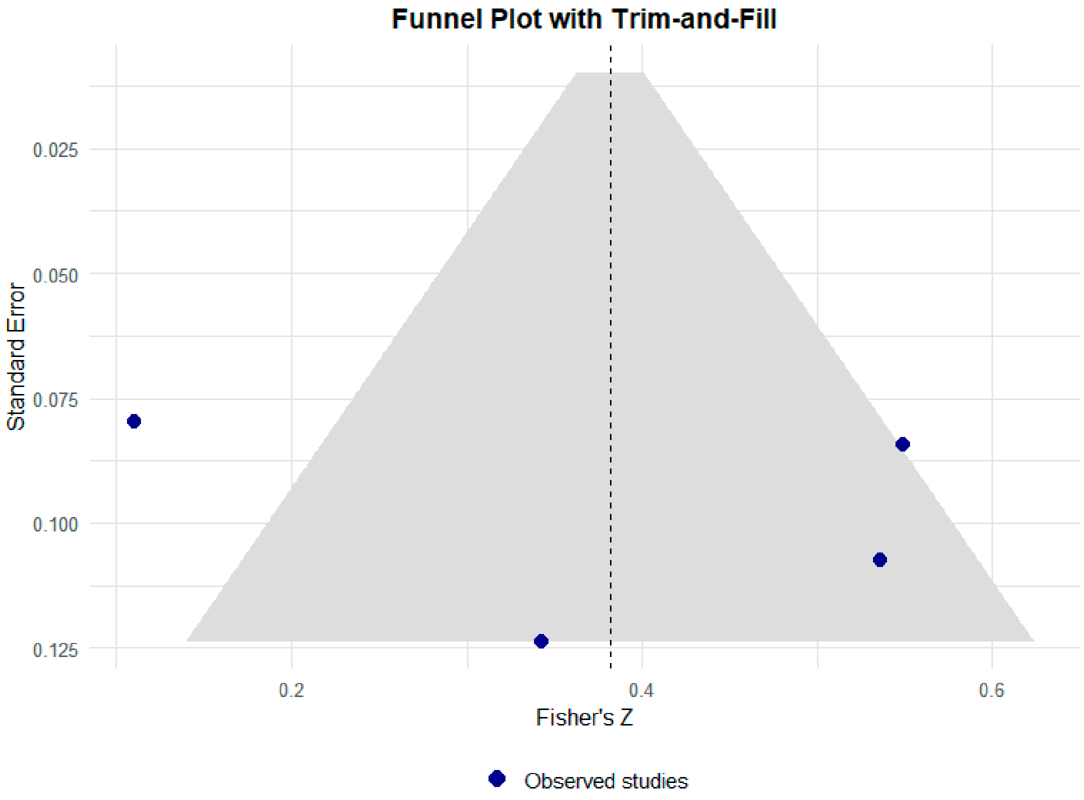

Figure S17. Funnel plot with trim-and-fill method assessing publication bias in the correlation between mammary adipocyte diameter and aromatase expression. Funnel plot applying the trim-and-fill procedure under a random-effects model. No imputed studies were identified, confirming the absence of publication bias.
